# Rapid screening mutations of first-line-drug-resistant genes in *Mycobacterium tuberculosis* strains by allele-specific real-time quantitative PCR

**DOI:** 10.7717/peerj.6696

**Published:** 2019-04-01

**Authors:** Pengpeng Yang, Yuzhu Song, Xueshan Xia, A-Mei Zhang

**Affiliations:** Faculty of Life Science and Technology, Kunming University of Science and Technology, Kunming, China

**Keywords:** AS-qPCR, Drug-resistant mutations, Specificity and sensitivity, TB

## Abstract

Tuberculosis (TB) is a worldwide health, economic, and social burden, especially in developing countries. Drug-resistant TB is the most serious type of this burden. Thus, it is necessary to screen drug-resistant mutations by using a simple and rapid detection method. A total of 32 pairs of allele-specific PCR (AS-PCR) primers were designed to screen mutation and/or wild-type alleles of 16 variations in four first-line drug-resistant genes (*katG*, *rpoB*, *rpsL*, and *embB*) of TB strains. A pair of primers was designed to amplify 16S rRNA gene and to verify successful amplification. Subsequently, we tested the specificity and sensitivity of these AS-PCR primers. The optimized condition of these AS-PCR primers was first confirmed. All mutations could be screened in general AS-PCR, but only 13 of 16 variations were intuitively investigated by using real-time quantitative PCR (qPCR) and AS-PCR primers. The results of specificity assay suggested that the AS-PCR primers with mutation and/or wildtype alleles could successfully amplify the corresponding allele under optimized PCR conditions. The sensitivity of nine pairs of primers was 500 copy numbers, and the other seven pairs of primers could successfully amplify correct fragments with a template comprising 10^3^ or 10^4^ copy numbers template. An optimized AS-qPCR was established to screen drug-resistant mutations in TB strains with high specificity and sensitivity.

## Introduction

Tuberculosis (TB) is a disease with high prevalence and mortality rate. It is caused by *Mycobacterium tuberculosis* (*M. tuberculosis*) infection. One-third of the world population is infected with *M. tuberculosis*, and 5% of infected people developed into TB in their lifetime (Comstock, Livesay & Woolpert, 1974; Koul et al., 2011). In 2017, 10.1 million people suffered from TB, among which 1.6 million people died. Though morbidity and mortality of TB gradually decreased with the appearance of anti-TB drugs, the mutation rate of drug-resistant genes in *M. tuberculosis* seemed to increase recently. The cost of TB treatment and research reached $ 10.4 billion in 2018. Half of this amount was used to treat drug-resistant TB (DR-TB) patients (WHO, 2018).

In the middle of 20th century, DR-TB strains were reported for the first time (Crofton & Mitchison, 1948). Unfortunately, the researchers did not focus on this phenomena at that time (Zignol et al., 2016). Recently, the number of patients with DR-TB, especially those with multiple drug-resistant TB (MDR-TB), seriously increased. The numbers of patients with MDR-TB reached 160,684 in 2017. A total of 10,800 cases of extensive drug-resistant TB (XDR-TB) were reported by 77 countries, and 88% XDR-TB cases were from European and South-East Asia regions (WHO, 2018). Due to abuse of antibiotics and environmental disruption, DR-TB strains have turned out to be barriers to TB treatment.

First-line anti-TB drugs, including isoniazid, rifampicin (RIF), streptomycin (SM), ethambutol (EMB), and pyrazinamide (PZA), are still widely used in clinics. Inevitably, drug-resistant genes exist in *M. tuberculosis*, thereby allowing it to resist first-line anti-TB drugs. *katG* and *inhA* genes are two common candidate genes in isoniazid (INH) -resistant TB strains. Mutations in other genes, such as *sigI*, *ndh*, and others, also reportedly lead to INH-resistance in TB (Guo et al., 2006). Similarly, *rpoB*, *embB*, *rpsL*, and *pncA* were major drug-resistant genes for RIF-, EMB-, SM-, and PZA- resistant TB strains (Lee et al., 2012; Sandy et al., 2002; Zhang & Yew, 2009). Although the mutant spectra showed distinction in different countries, there were some hotspot mutations in these candidate genes. The mutation at codon 315 of the *katG* gene was the most popular INH-resistant mutation, and more than 50% of INH-resistant mutations located in this codon (Afanas’ev et al., 2007; Yuan et al., 2012). Most of RIF-resistant mutations located in hotspot region of the *rpoB* gene, but the mutant frequency varied from 75% to 90% in different countries (Franco-Sotomayor et al., 2018; Thirumurugan et al., 2015). Mutations at codon 43 and 88 were two common SM-resistant mutations in the *rpsL* gene, and over 65% SM-resistant TB strains were caused by these two mutations (Tudo et al., 2010; Zhao et al., 2015). About 70% of EMB-resistant TB strains had mutations located at codon 306, 406, or 497 in the *embB* gene (Brossier et al., 2015). Thus, rapid screening for these mutations was necessary.

Long-time and inappropriate drug usage could lead to drug-resistance. Thus, rapid and convenient diagnosis of DR-TB patients is necessary for further effective treatment. Based on the drug-resistant mutations (including hotspot and rare mutations) in our previous study (Li et al., 2017), we established an optimized allele-specific real-time quantitative PCR (AS-qPCR) method to screen mutations in DR-TB strains rapidly and with high sensitivity and specificity.

## Materials and Methods

### *Mycobacterium tuberculosis* strains collection and DNA extraction

Drug-resistant *M. tuberculosis* strains were collected and cultured in Lowenstein-Jensen medium by doctors in Kunming Third People’s Hospital. Drug susceptibility testing (DST) was performed by using the following drugs: INH, 0.2 mg/L; RIF, 40 mg/L; SM, 4.0 mg/L; EMB, 2.0 mg/L; and PZA, 100 mg/L. G^+^ Bacteria Genomic DNA Kit (ZOMANBIO, Beijing, China) was used to extract genomic DNA from *M. tuberculosis* strains according to the manufacturer’s instructions. This study was approved by the institutional review board of Kunming University of Science and Technology (Approval No. 2014SK027).

### Primer design and AS-PCR optimization

A total of 32 pairs of primers for AS-PCR were designed to screen the 16 variations (including mutation and wild-type alleles), which were located in four genes (*katG*, *embB*, *rpsL*, and *pncA*), by using Oligo Primer Analysis Software v.7 (Table S1). To strengthen the specificity of primers, a non-complementary nucleotide in 3′ end of the allele-specific primer was factitiously changed and marked in red in Table S1. One pair of inner control primers (16S 915-F/16S 1018-R) was used to control and identify PCR quantification (Table S1).

PCR was performed in 20 µL reaction volume and involved 30 ng of genomic DNA, 10 µL 2× TSINGKE^™^ Master Mix (including one U DNA polymerase, 1.5 mM MgCl_2_, 50 mM KCl, and 100 mM dNTP) (TSINGKE, Beijing, China) or ChamQ^™^ SYBR qPCR Master Mix (Vazyme, Nanjing, China), and 0.5 µM each primer (including AS-PCR primers and internal control primers). After optimization, we used the following PCR condition: one cycle of 95 °C for 3 min; 35 cycles of 95 °C for 30 s, optimized temperature for 30 s (Table S1), 72 °C for 10 s; and one extension cycle of 72 °C for 5 min.

### Plasmids construction

Five pairs of primers (Table S2) were designed to amplify fragments containing all mutations. PCR amplification products were ligated into the pClone 007 Blunt Simple Vector by using pClone 007 Vector Kit (TSINGKE, Beijing, China). All constructed plasmids were identified to carry the specific mutation or wildtype allele by sequencing. Plasmid extraction small Kit (TIANGEN, Beijing, China) was used to purify the plasmids.

### Specificity and sensitivity tests

We amplified 14 wildtype TB samples (identified by sequencing) and all templets of each mutation to test the specificity of AS-PCR by using both wildtype and mutation primers. If there was only one sample with a certain mutation, we duplicated the AS-PCR by using the same mutation sample. The sensitivity results were determined based on the appearance and intensity of the products on the agarose gel. Moreover, wildtype AS-PCR primers were also used to amplify plasmids with corresponding mutations. After qualifying the plasmids, we diluted plasmids to 10^4^, 10^3^, and 5 × 10^2^ copy numbers to achieve a sensitive assay.

### AS real-time quantitative PCR

According to the results of optimized AS-PCR, we performed AS-qPCR assays, in order to directly detect the products and determine the mutation or wild type allele. PCR was performed in 20 µL reaction volume and involved 30 ng of genomic DNA, ChamQTM SYBR qPCR Master Mix (Vazyme, Nanjing, China), and 0.3 µM each primer (including mutation and wild type AS-PCR primers of each allele) on Takara Thermal Cycler Dice Real Time System TP800 (TaKaRa, Kusatsu, Japan).

## Results

**Figure 1:**
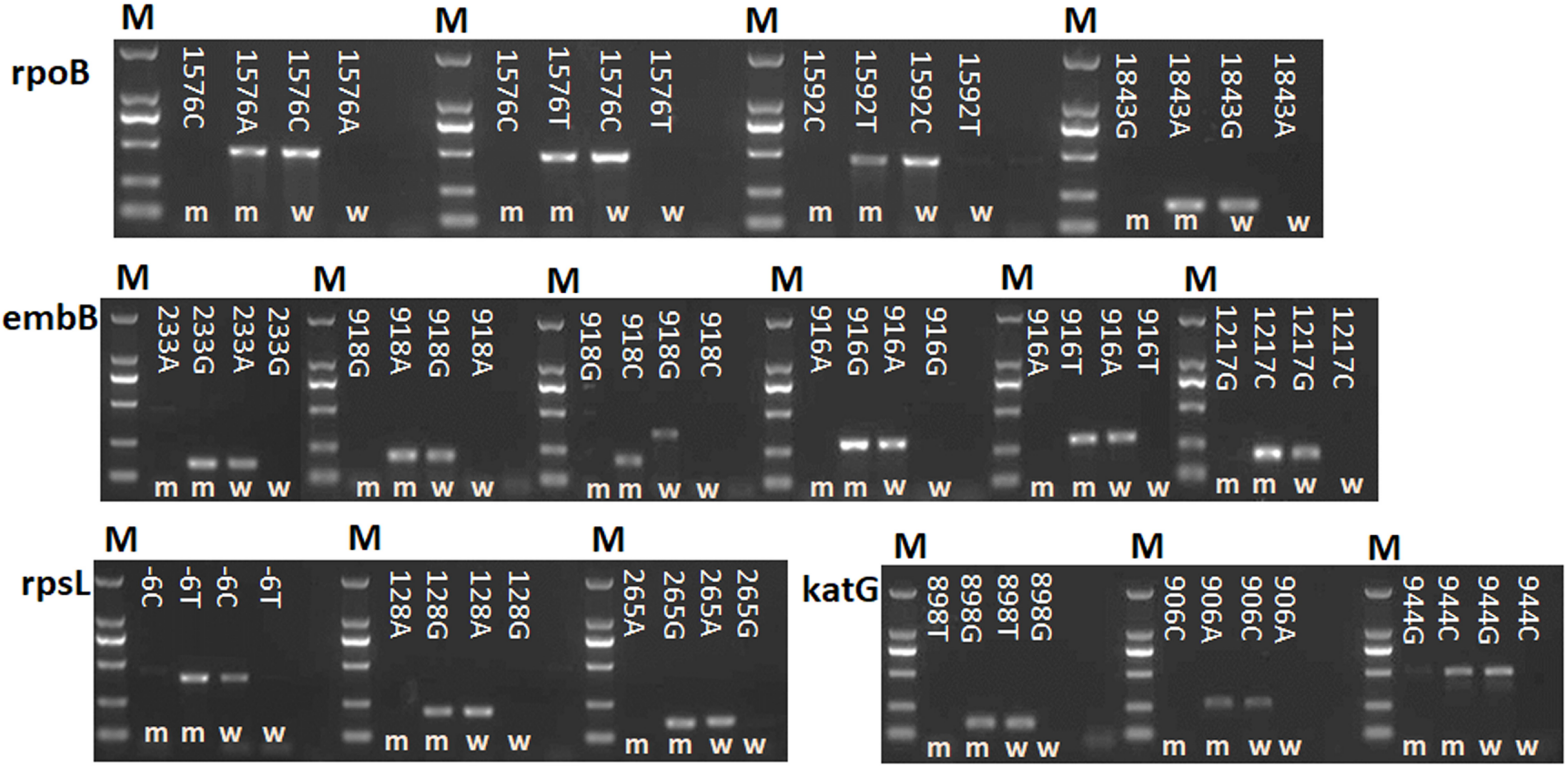
Detection of 16 mutations in four first-line drug-resistance genes by using AS-PCR. M means DNA marker DL2000; m and w mean the AS-PCR primers were used to screen mutation and wildtype alleles, respectively. NC means negative control.

**Figure 2:**
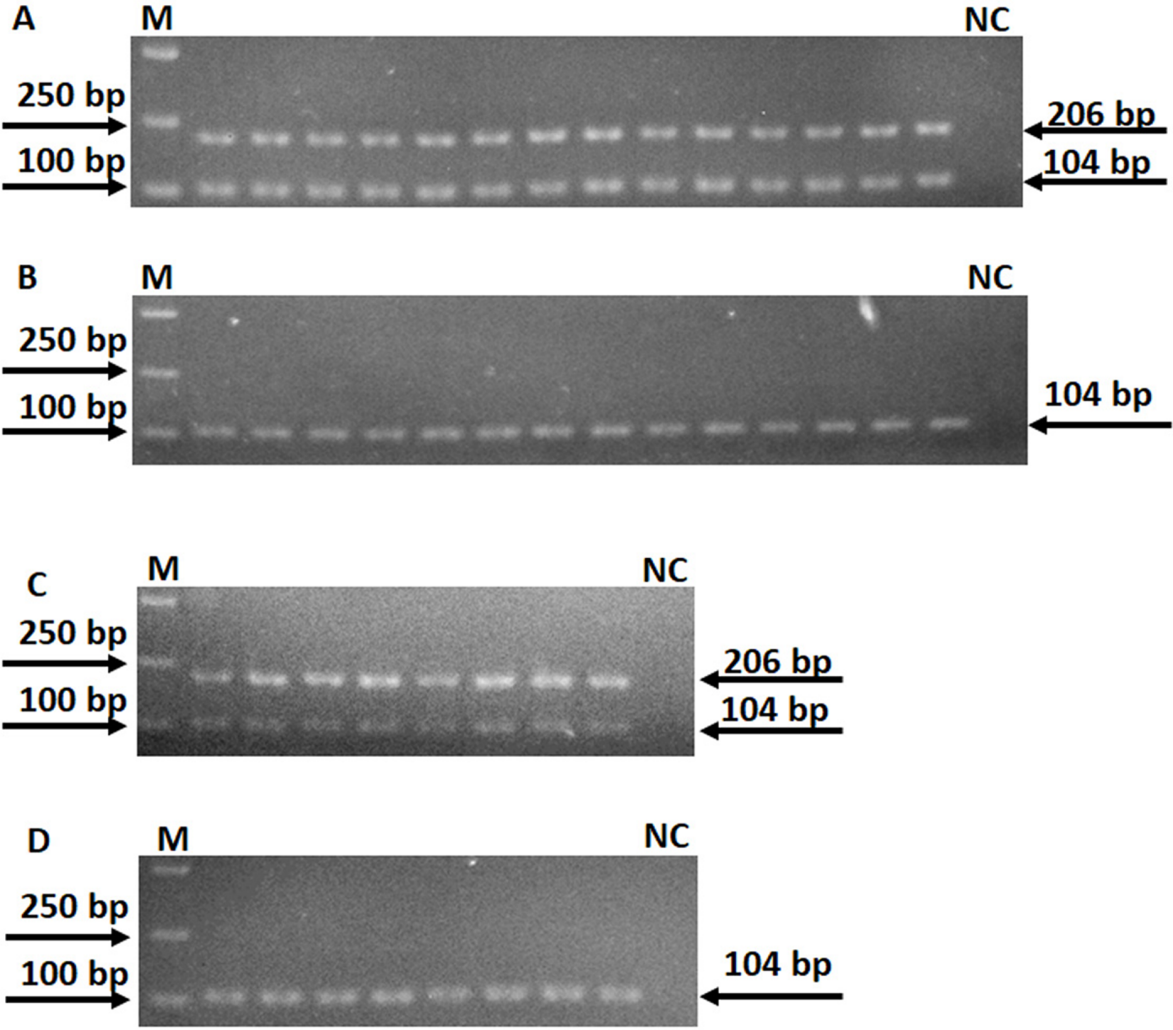
Specificity of AS-PCR primers for A128G in the *rpsL* gene. (A) PCR were performed by using 14 DNA templates with allele A and wildtype AS-PCR primers. (B) PCR were performed by using 14 DNA templates with allele A and mutation AS-PCR primers. (C) PCR were performed by using eight DNA templates with allele G and mutation AS-PCR primers. (D) PCR were performed by using eight DNA templates with allele G and wildtype AS-PCR primers. Fragments at 206 bp mean the specific product by AS-PCR primers; fragments at 104 bp mean the inner control product by inner primers; NC means negative control.

A total of 16 point mutations in four first-line drug-resistant genes were used to establish AS-PCR detecting method, which were identified in 57 drug-resistant *M. tuberculosis* strains by sequencing in our previous study (Li et al., 2017). After optimizing the conditions of PCR with these primers, we obtained concordant results by sequencing, that is, the correct bands were successfully amplified by using mutant AS-PCR primers and mutation template but not wild-type samples, and vice versa (Fig. 1). The specificity test results suggested all primers in this study could identify the mutation or wildtype alleles with high fidelity (Fig. S1). As shown in Fig. 2A 206 bp fragment could be amplified by using samples with wild-type *rpsL* gene and primers *RpsL* 128A-F/*RpsL* 128-R (the primer for wild-type allele A at amino acid codon 128). However, this amplicon did not exist when samples with *rpsL* 128G were used. On the contrary, the primers *RpsL* 128G-F/*RpsL* 128-R (for mutant allele G) could amplify a 206 bp fragment when the samples carried the mutation allele 128 G in the *rpsL* gene. The presentation of the 104 bp inner control fragment suggested a successful amplification.

**Figure 3:**
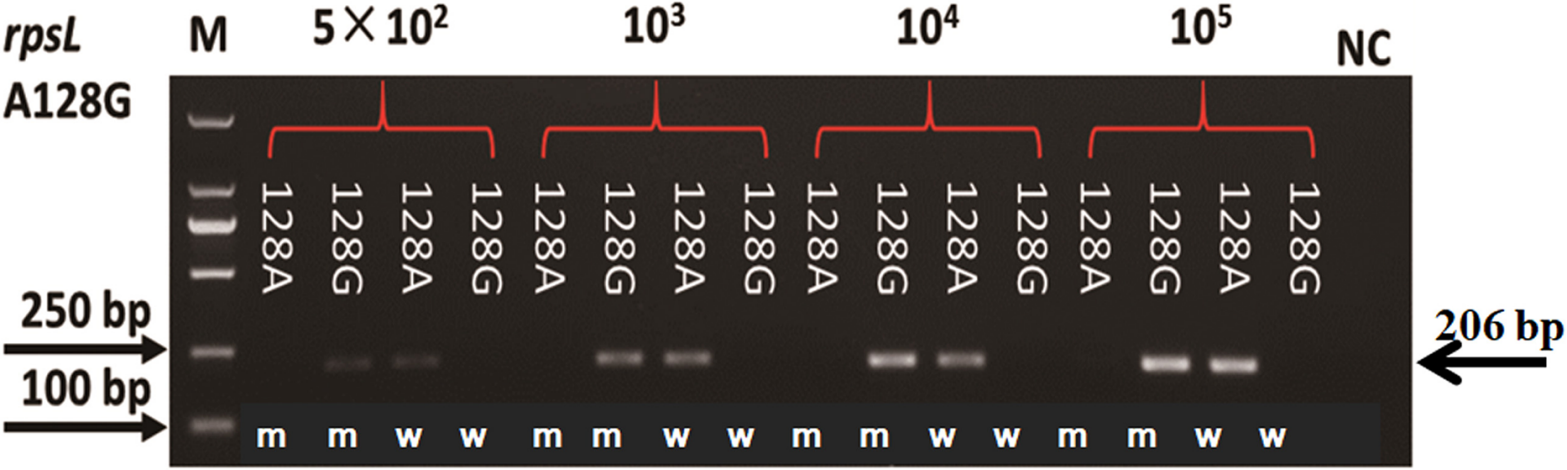
Sensitivity of AS-PCR primers for A128G in the *rpsL* gene. M means DNA marker DL2000; m and w mean the AS-PCR primers were used to screen mutation and wildtype alleles, respectively.

Plasmids with mutation and wildtype alleles were constructed to evaluate the sensitivity of AS-PCR primers. As shown in Fig. 3, the sensitivity of primers for amino acid codon 128 in the *rpsL* gene was estimated by using a plasmid with 10^4^, 10^3^, and 5 × 10^2^ copy numbers. When the copy number of the plasmids was 5 × 10^2^, we could visualize a faint band. However, the bands were obvious and clear when we increased the plasmids to 10^3^ or 10^4^ copy numbers. The sensitivity of all 32 pairs of primers were tested, and half of these primers could amplify an observable band when the plasmid copy number was 5 × 10^2^ (Fig. S2). However, the others needed more copy numbers (10^3^ or 10^4^, Fig. S2; Table S1).

**Figure 4:**
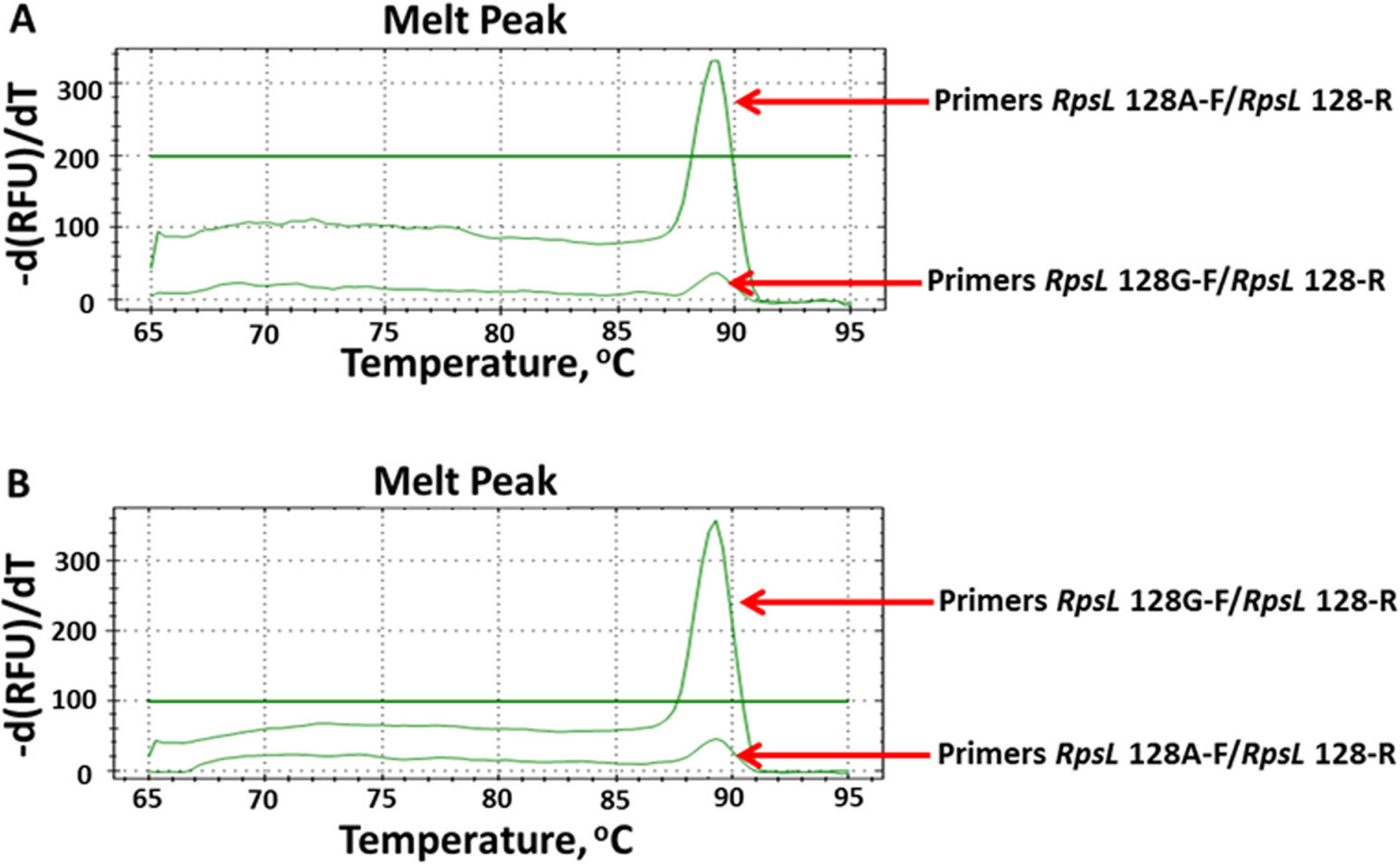
Detecting allele type of A128G in the *rpsL* genes by using AS-PCR primers and real-time quantitative PCR. (A) Melt curves of product by using DNA template with allele A. (B) Melt curves of product by using DNA template with allele G.

To directly and rapidly investigate the testing results of AS-PCR, we combined AS-PCR primers and qPCR for subsequent observation. By observing the melt-curve of these products, we determined whether mutations existed. We firstly defined the baseline at 100 relative fluorescence units/min as the detecting level. According to this standard, we tested all 16 mutations by using AS-qPCR. However, only 13 mutations were rapidly and directly genotyped (Fig. S3). As shown in Fig. 4, we could rapidly identify the allele at 128 nucleotide in the *rpsL* gene. By using the optimized AS-qPCR, we rapidly distinguished the mutation and/or wild-type allele in one PCR reaction with two pairs of primers.

## Discussion

China is among the top 22 countries with the highest burden of TB and with the second highest burden of DR-TB. About 120,000 persons developed into TB in China each year (Du et al., 2017; Zhao et al., 2012). Many factors could lead to DR-TB, such as contaminative environment, drug abuse, long-time therapy, and host genetic factors. Some TB strains might change to drug-resistance or solo DR-TB might develop to XDR-TB after the long-term treatment of TB patients. Thus, it was important and necessary to rapidly diagnose DR-TB, especially for first-line DR-TB. Point mutations of drug-resistant genes were common reasons for the development of first-line DR-TB strains. Mutations in the *katG* and/or *inhA* gene were the two main causes of INH-resistant TB. About 51% and 10% isoniazid-resistant TB strains were caused by mutations in the *katG* and *inhA* genes, respectively (Guo et al., 2006). Mutations in the *rpoB* gene, *rpsL* gene, *embB gene*, and *pncA* gene were the main factors for RIF-resistant, SM-resistant, EMB-resistant, and PZA-resistant TB strains, respectively (Brossier et al., 2015; Scorpio & Zhang, 1996; Stoffels et al., 2012; Villellas et al., 2013). Furthermore, some hotspot mutations still exist in these drug-resistant genes (Banerjee et al., 1994; Dalla Costa et al., 2009; Lee et al., 2012; Seifert et al., 2015; Waagmeester, Thompson & Reyrat, 2005). Most commercialized detecting kits for DR-TB strains only contained hotspot mutations. Hence, some rare mutations might be missed. In our previous study, we identified some mutations in these genes of Yunnan DR-TB strains, and some of them were hotspot mutations and others were rare mutations (Li et al., 2017). Because no hotspot mutation has been found in the *pncA* gene, 18 mutations of the other four genes (including *katG*, *rpoB*, *rpsL*, and *embB* genes) were used as candidate mutations in this study. Unfortunately, two mutations, including G1388T (at codon 463) in the *katG* gene and A1490G (at codon 497) in the *embB* gene, could not be genotyped by using AS-PCR. Thus, it seemed that not all drug-resistant mutations could be well detected by using AS-PCR.

Drug susceptibility testing is the classic method and the “gold standard” for the evaluation of DR-TB strains (Ahmad & Mokaddas, 2009). Until now, DST is still widely used in laboratory and hospital, but methods to screen mutations of drug-resistant genes were speedily developed after the invention of PCR. Multi-fluorescence qPCR is one of the most common methods to detect RIF- and INH-resistant *M. tuberculosis* (Peng et al., 2016). Other technologies, such as whole-genome sequencing (WGS) (Pankhurst et al., 2016), high-resolution melt, PCR-single strand conformation polymorphism, and oligonucleotide microarrays (Caoili et al., 2006; Herrmann et al., 2006; Pietzka et al., 2009; Traore et al., 2006), have also been widely used to screen the mutations of candidate genes in DR-TB strains. However, these technologies have their advantages and shortcomings. For example, high quality and various mutation types could be detected using WGS, but the expensive equipment and reagent limited its usage.

Since AS-PCR was first reported in 1989 (Newton et al., 1989), it has been widely used to screen single nucleotide polymorphisms and mutations. Although AS-PCR is considered as low specificity and sensitivity (Sharma et al., 2016), Onseedaeng & Ratthawongjirakul (2016) identified mutations of the *gyrA* and *parC* gene in *Escherichia coli* with high sensitivity and specificity by using AS-PCR. Due to its simple operation, low cost, and relatively high specificity and sensitivity, we successfully used AS-PCR to screen 16 mutations in four first-line DR-genes. After optimizing reaction conditions, we successfully amplified the corresponding bands by using AS-PCR primers with high specificity. Most of these primers detected the corresponding mutations when the DNA template reached 500 copy numbers. All these results suggested that optimized AS-qPCR could be used to screen mutations of drug-resistant genes in TB strains with higher specificity and sensitivity. A limitation of this current study was the small size of the number of drug-resistant mutations. One reason was that no more TB-strains with other drug-resistant mutations were obtained in this experiment; another reason was several drug-resistant mutations could not be well genotyped by using the AS-PCR or AS-qPCR method. In further studies, we should collect more DR-TB strains from various regions and further optimize AS-PCR condition for rapid screening.

## Conclusion

In summary, we established an optimized AS-qPCR method to screen mutations in four first-line drug-resistant genes of *M. tuberculosis* with relatively high specificity and sensitivity. This AS-qPCR could be widely used in the future to rapidly screen mutations in DR-TB strains.

## Supplemental Information

10.7717/peerj.6696/supp-1 Supplemental Information 1 Figure S1. Electrophoresis map of specific tests.

10.7717/peerj.6696/supp-2 Supplemental Information 2 Figure S2. Electrophoresis map of sensitivity tests.

10.7717/peerj.6696/supp-3 Supplemental Information 3 Figure S3. Melt curves map of real-time quantitative PCR.

10.7717/peerj.6696/supp-4 Supplemental Information 4 Table S1. Information of AS-PCR primers.

10.7717/peerj.6696/supp-5 Supplemental Information 5 Table S2. Information for primers used to construct plasmids.

## Additional Information and Declarations

### Data Availability

The following information was supplied regarding data availability: Raw data is available in the Supplemental Files.

## References

[ref-1] Afanas’ev MV, Ikryannikova LN, Il’ina EN, Sidorenko SV, Kuz’min AV, Larionova EE, Smirnova TG, Chernousova LN, Kamaev EY, Skorniakov SN, Kinsht VN, Cherednichenko AG, Govorun VM (2007). Molecular characteristics of rifampicin- and isoniazid-resistant *Mycobacterium tuberculosis* isolates from the Russian Federation. Journal of Antimicrobial Chemotherapy.

[ref-2] Ahmad S, Mokaddas E (2009). Recent advances in the diagnosis and treatment of multidrug-resistant tuberculosis. Respiratory Medicine.

[ref-3] Banerjee A, Dubnau E, Quemard A, Balasubramanian V, Um KS, Wilson T, Collins D, De Lisle G, Jacobs WR (1994). inhA, a gene encoding a target for isoniazid and ethionamide in *Mycobacterium tuberculosis*. Science.

[ref-4] Brossier F, Sougakoff W, Bernard C, Petrou M, Adeyema K, Pham A, De la Breteque DA, Vallet M, Jarlier V, Sola C, Veziris N (2015). Molecular analysis of the *embCAB* locus and *embR* gene involved in ethambutol resistance in clinical isolates of Mycobacterium tuberculosis in France. Antimicrobial Agents and Chemotherapy.

[ref-5] Caoili JC, Mayorova A, Sikes D, Hickman L, Plikaytis BB, Shinnick TM (2006). Evaluation of the TB-Biochip oligonucleotide microarray system for rapid detection of rifampin resistance in *Mycobacterium tuberculosis*. Journal of Clinical Microbiology.

[ref-6] Comstock GW, Livesay VT, Woolpert SF (1974). The prognosis of a positive tuberculin reaction in childhood and adolescence. American Journal of Epidemiology.

[ref-7] Crofton J, Mitchison DA (1948). Streptomycin resistance in pulmonary tuberculosis. BMJ.

[ref-8] Dalla Costa ER, Ribeiro MO, Silva MSN, Arnold LS, Rostirolla DC, Cafrune PI, Espinoza RC, Palaci M, Telles MA, Ritacco V, Suffys PN, Lopes ML, Campelo CL, Miranda SS, Kremer K, Da Silva PE, De Souza Fonseca L, Ho JL, Kritski AL, Rossetti MLR (2009). Correlations of mutations in katG, oxyR-ahpC and inhA genes and in vitro susceptibility in *Mycobacterium tuberculosis* clinical strains segregated by spoligotype families from tuberculosis prevalent countries in South America. BMC Microbiology.

[ref-9] Du J, Pang Y, Ma Y, Mi F, Liu Y, Li L (2017). Prevalence of tuberculosis among health care workers in tuberculosis specialized hospitals in China. Journal of Occupational Health.

[ref-10] Franco-Sotomayor G, Garzon-Chavez D, Leon-Benitez M, De Waard JH, Garcia-Bereguiain MA (2018). A first insight into the *katG* and *rpoB* gene mutations of multidrug-resistant *My*cobacterium tuberculosis strains from ecuador. Microbial Drug Resistance.

[ref-11] Guo H, Seet Q, Denkin S, Parsons L, Zhang Y (2006). Molecular characterization of isoniazid-resistant clinical isolates of *Mycobacterium tuberculosis* from the USA. Journal of Medical Microbiology.

[ref-12] Herrmann MG, Durtschi JD, Bromley LK, Wittwer CT, Voelkerding KV (2006). Amplicon DNA melting analysis for mutation scanning and genotyping: cross-platform comparison of instruments and dyes. Clinical Chemistry.

[ref-13] Koul A, Arnoult E, Lounis N, Guillemont J, Andries K (2011). The challenge of new drug discovery for tuberculosis. Nature.

[ref-14] Lee J-H, Ammerman NC, Nolan S, Geiman DE, Lun S, Guo H, Bishai WR (2012). Isoniazid resistance without a loss of fitness in *Mycobacterium tuberculosis*. Nature Communications.

[ref-15] Li D, Song Y, Zhang C-L, Li X, Xia X, Zhang A-M (2017). Screening mutations in drug-resistant Mycobacterium tuberculosis strains in Yunnan, China. Journal of Infection and Public Health.

[ref-16] Newton CR, Graham A, Heptinstall LE, Powell SJ, Summers C, Kalsheker N, Smith JC, Markham AF (1989). Analysis of any point mutation in DNA. The amplification refractory mutation system (ARMS). Nucleic Acids Research.

[ref-17] Onseedaeng S, Ratthawongjirakul P (2016). Rapid detection of genomic mutations in *gyrA* and *parC* genes of *Escherichia coli* by multiplex allele specific polymerase chain reaction. Journal of Clinical Laboratory Analysis.

[ref-18] Pankhurst LJ, Del Ojo Elias C, Votintseva AA, Walker TM, Cole K, Davies J, Fermont JM, Gascoyne-Binzi DM, Kohl TA, Kong C, Lemaitre N, Niemann S, Paul J, Rogers TR, Roycroft E, Smith EG, Supply P, Tang P, Wilcox MH, Wordsworth S, Wyllie D, Xu L, Crook DW, COMPASS-TB Study Group (2016). Rapid, comprehensive, and affordable mycobacterial diagnosis with whole-genome sequencing: a prospective study. Lancet Respiratory Medicine.

[ref-19] Peng J, Yu X, Cui Z, Xue W, Luo Z, Wen Z, Liu M, Jiang D, Zheng H, Wu H, Zhang S, Li Y (2016). Multi-fluorescence real-time PCR assay for detection of RIF and INH resistance of *M. tuberculosis*. Frontiers in Microbiology.

[ref-20] Pietzka AT, Indra A, Stoger A, Zeinzinger J, Konrad M, Hasenberger P, Allerberger F, Ruppitsch W (2009). Rapid identification of multidrug-resistant *Mycobacterium tuberculosis* isolates by rpoB gene scanning using high-resolution melting curve PCR analysis. Journal of Antimicrobial Chemotherapy.

[ref-21] Sandy J, Mushtaq A, Kawamura A, Sinclair J, Sim E, Noble M (2002). The structure of arylamine N-acetyltransferase from Mycobacterium smegmatis—an enzyme which inactivates the anti-tubercular drug, isoniazid. Journal of Molecular Biology.

[ref-22] Scorpio A, Zhang Y (1996). Mutations in pncA, a gene encoding pyrazinamidase/nicotinamidase, cause resistance to the antituberculous drug pyrazinamide in tubercle bacillus. Nature Medicine.

[ref-23] Seifert M, Catanzaro D, Catanzaro A, Rodwell TC (2015). Genetic mutations associated with isoniazid resistance in *Mycobacterium tuberculosis*: a systematic review. PLOS ONE.

[ref-24] Sharma D, Lather M, Dykes CL, Dang AS, Adak T, Singh OP (2016). Disagreement in genotyping results of drug resistance alleles of the Plasmodium falciparum dihydrofolate reductase (Pfdhfr) gene by allele-specific PCR (ASPCR) assays and Sanger sequencing. Parasitology Research.

[ref-25] Stoffels K, Mathys V, Fauville-Dufaux M, Wintjens R, Bifani P (2012). Systematic analysis of pyrazinamide-resistant spontaneous mutants and clinical isolates of *Mycobacterium tuberculosis*. Antimicrobial Agents and Chemotherapy.

[ref-26] Thirumurugan R, Kathirvel M, Vallayyachari K, Surendar K, Samrot AV, Muthaiah M (2015). Molecular analysis of rpoB gene mutations in rifampicin resistant *Mycobacterium tuberculosis* isolates by multiple allele specific polymerase chain reaction in Puducherry, South India. Journal of Infection and Public Health.

[ref-27] Traore H, Van Deun A, Shamputa IC, Rigouts L, Portaels F (2006). Direct detection of *Mycobacterium tuberculosis* complex DNA and rifampin resistance in clinical specimens from tuberculosis patients by line probe assay. Journal of Clinical Microbiology.

[ref-28] Tudo G, Rey E, Borrell S, Alcaide F, Codina G, Coll P, Martin-Casabona N, Montemayor M, Moure R, Orcau A, Salvado M, Vicente E, Gonzalez-Martin J (2010). Characterization of mutations in streptomycin-resistant *Mycobacterium tuberculosis* clinical isolates in the area of Barcelona. Journal of Antimicrobial Chemotherapy.

[ref-29] Villellas C, Aristimuno L, Vitoria M-A, Prat C, Blanco S, Garcia De Viedma D, Dominguez J, Samper S, Ainsa JA (2013). Analysis of mutations in streptomycin-resistant strains reveals a simple and reliable genetic marker for identification of the *Mycobacterium tuberculosis* Beijing genotype. Journal of Clinical Microbiology.

[ref-30] WHO (2018). Global tuberculosis report. http://www.who.int/tb/publications/factsheet_global.pdf?ua=1.

[ref-31] Waagmeester A, Thompson J, Reyrat J-M (2005). Identifying sigma factors in Mycobacterium smegmatis by comparative genomic analysis. Trends in Microbiology.

[ref-32] Yuan X, Zhang T, Kawakami K, Zhu J, Li H, Lei J, Tu S (2012). Molecular characterization of multidrug- and extensively drug-resistant *Mycobacterium tuberculosis* strains in Jiangxi, China. Journal of Clinical Microbiology.

[ref-33] Zhang Y, Yew WW (2009). Mechanisms of drug resistance in *Mycobacterium tuberculosis*. International Journal of Tuberculosis and Lung Disease.

[ref-34] Zhao L-L, Liu H-C, Sun Q, Xiao T-Y, Zhao X-Q, Li G-L, Zeng C-Y, Wan K-L (2015). Identification of mutations conferring streptomycin resistance in multidrug-resistant tuberculosis of China. Diagnostic Microbiology and Infectious Disease.

[ref-35] Zhao Y, Xu S, Wang L, Chin DP, Wang S, Jiang G, Xia H, Zhou Y, Li Q, Ou X, Pang Y, Song Y, Zhao B, Zhang H, He G, Guo J, Wang Y (2012). National survey of drug-resistant tuberculosis in China. New England Journal of Medicine.

[ref-36] Zignol M, Dean AS, Falzon D, Van Gemert W, Wright A, Van Deun A, Portaels F, Laszlo A, Espinal MA, Pablos-Méndez A, Bloom A, Aziz MA, Weyer K, Jaramillo E, Nunn P, Floyd K, Raviglione MC (2016). Twenty years of global surveillance of antituberculosis-drug resistance. New England Journal of Medicine.

