# Supplementary material for: Rapid screening mutations of first-line-drug-resistant genes in Mycobacterium tuberculosis strains by allele-specific real-time quantitative PCR
Source: PeerJ. 2019 Apr 1;7:e6696. doi: 10.7717/peerj.6696 (PMC6448557; doi:10.7717/peerj.6696)
Supplement: Supplemental Information 1 [file peerj-07-6696-s001.pdf]

katG
T898G

DNA template with allele T and wildtype AS-PCR primers

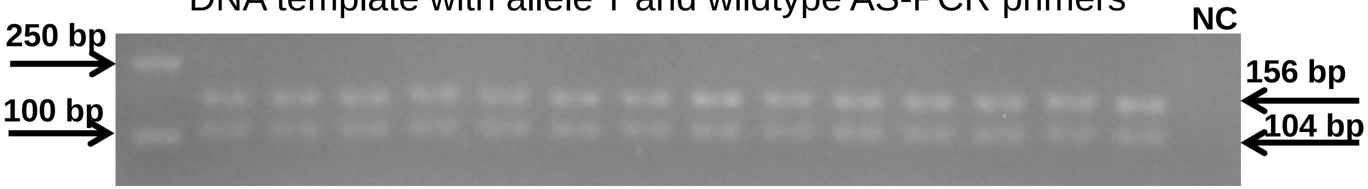

DNA template with allele T and mutation AS-PCR primers

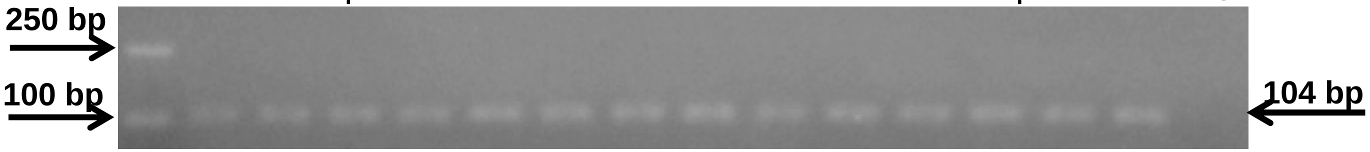

DNA template with allele G and mutation AS-PCR primers

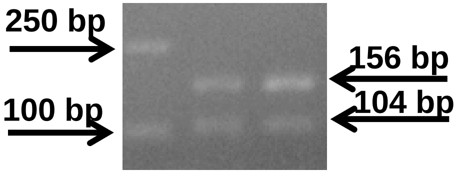

DNA template with allele G and wildtype AS-PCR primers

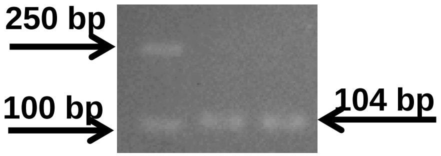

katG
C906A

DNA template with allele C and wildtype AS-PCR primers

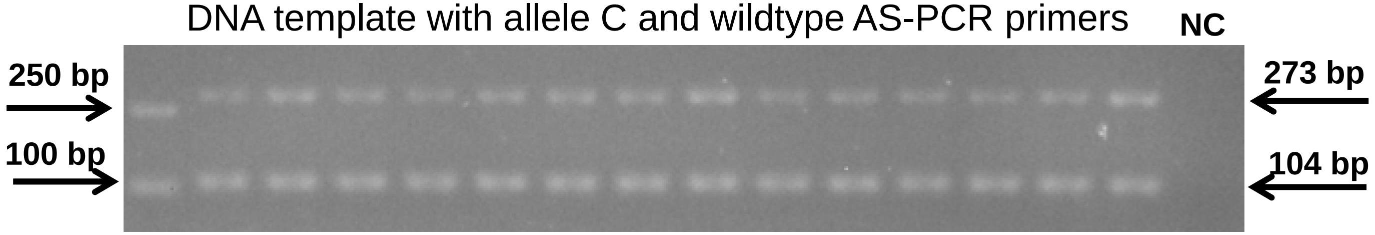

DNA template with allele C and mutation AS-PCR primers

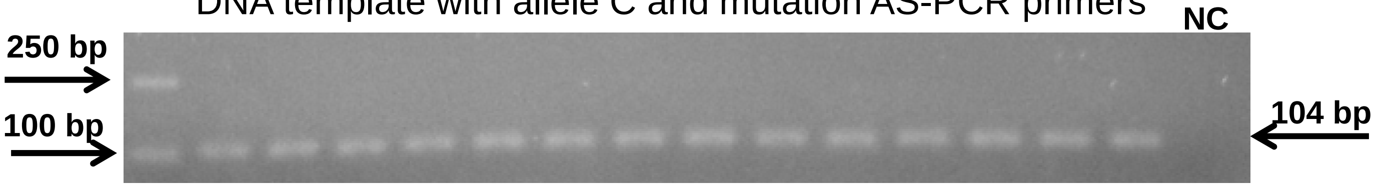

DNA template with allele A and mutation AS-PCR primers

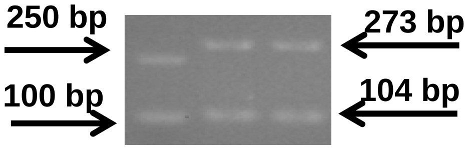

DNA template with allele A and wildtype AS-PCR primers

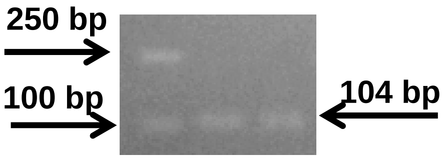

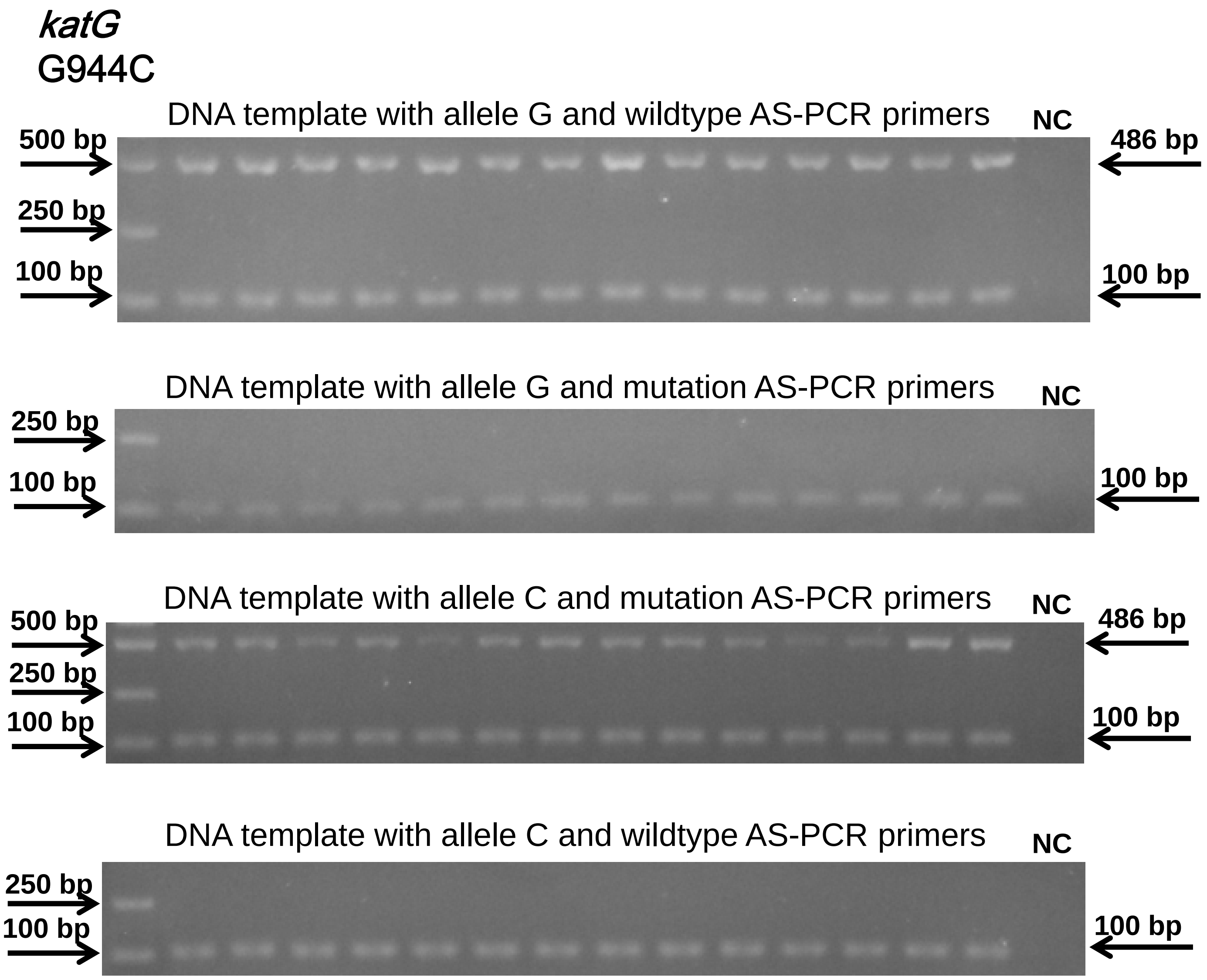
katG
G944C
DNA template with allele G and wildtype AS-PCR primers
NC
500 bp
250 bp
100 bp
486 bp
100 bp
DNA template with allele G and mutation AS-PCR primers
NC
250 bp
100 bp
100 bp
DNA template with allele C and mutation AS-PCR primers
NC
500 bp
250 bp
100 bp
486 bp
100 bp
DNA template with allele C and wildtype AS-PCR primers
NC
250 bp
100 bp
100 bp

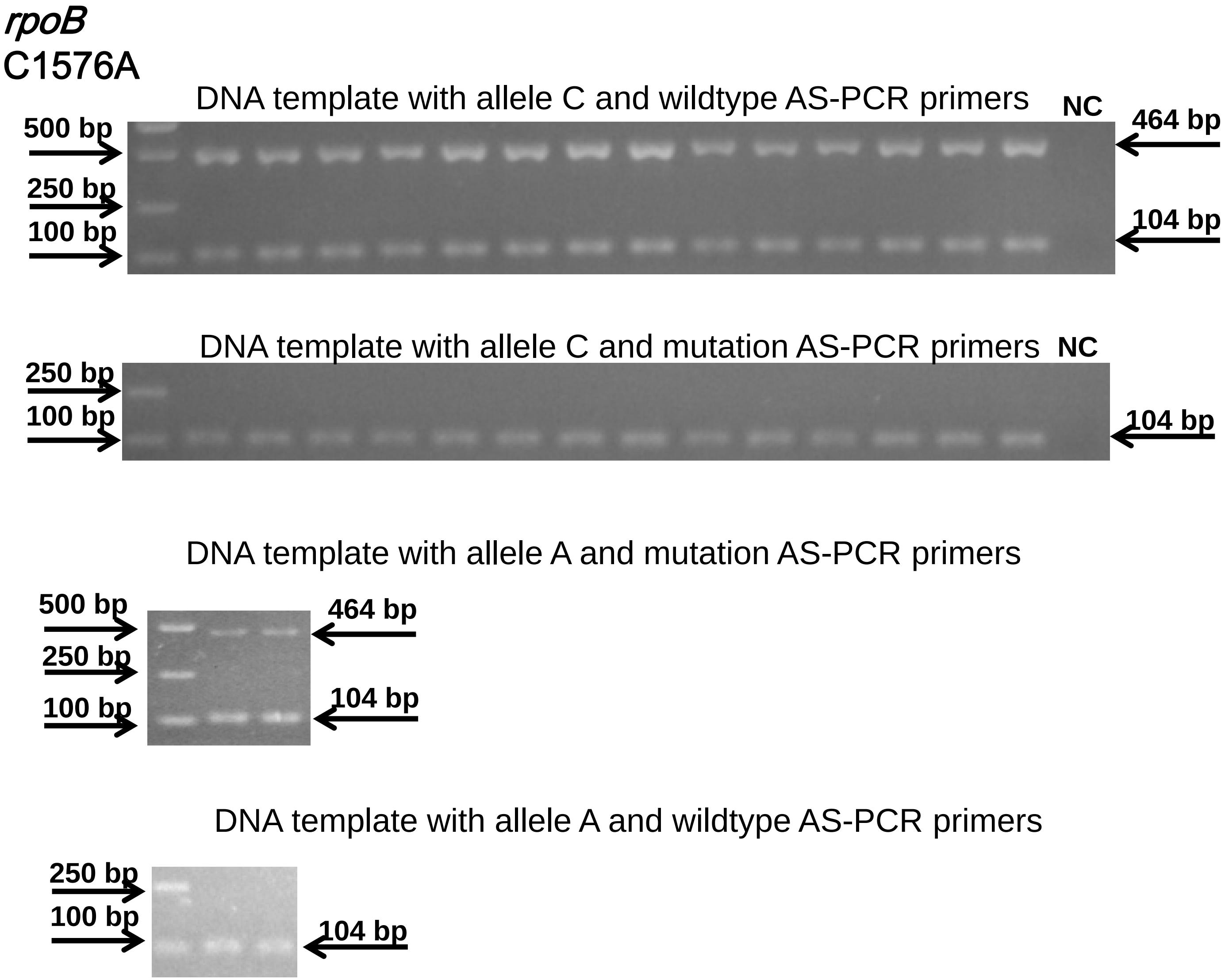
rpoB
C1576A
DNA template with allele C and wildtype AS-PCR primers
NC
500 bp
250 bp
100 bp
464 bp
104 bp
DNA template with allele C and mutation AS-PCR primers
NC
250 bp
100 bp
104 bp
DNA template with allele A and mutation AS-PCR primers
500 bp
250 bp
100 bp
464 bp
104 bp
DNA template with allele A and wildtype AS-PCR primers
250 bp
100 bp
104 bp

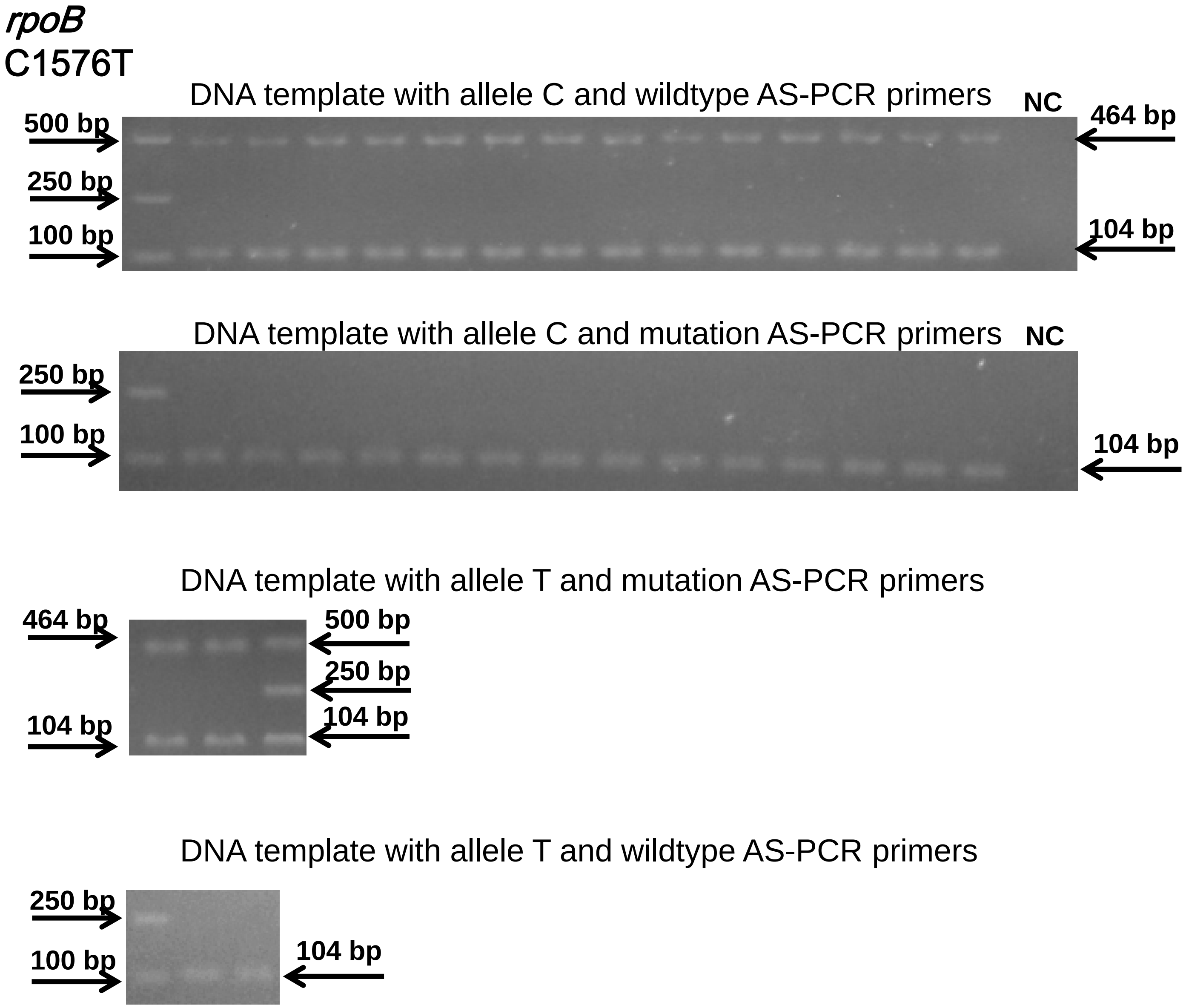

rpoB
C1576T
DNA template with allele C and wildtype AS-PCR primers
NC
500 bp
250 bp
100 bp
464 bp
104 bp
DNA template with allele C and mutation AS-PCR primers
NC
250 bp
100 bp
104 bp
DNA template with allele T and mutation AS-PCR primers
464 bp
104 bp
500 bp
250 bp
104 bp
DNA template with allele T and wildtype AS-PCR primers
250 bp
100 bp
104 bp

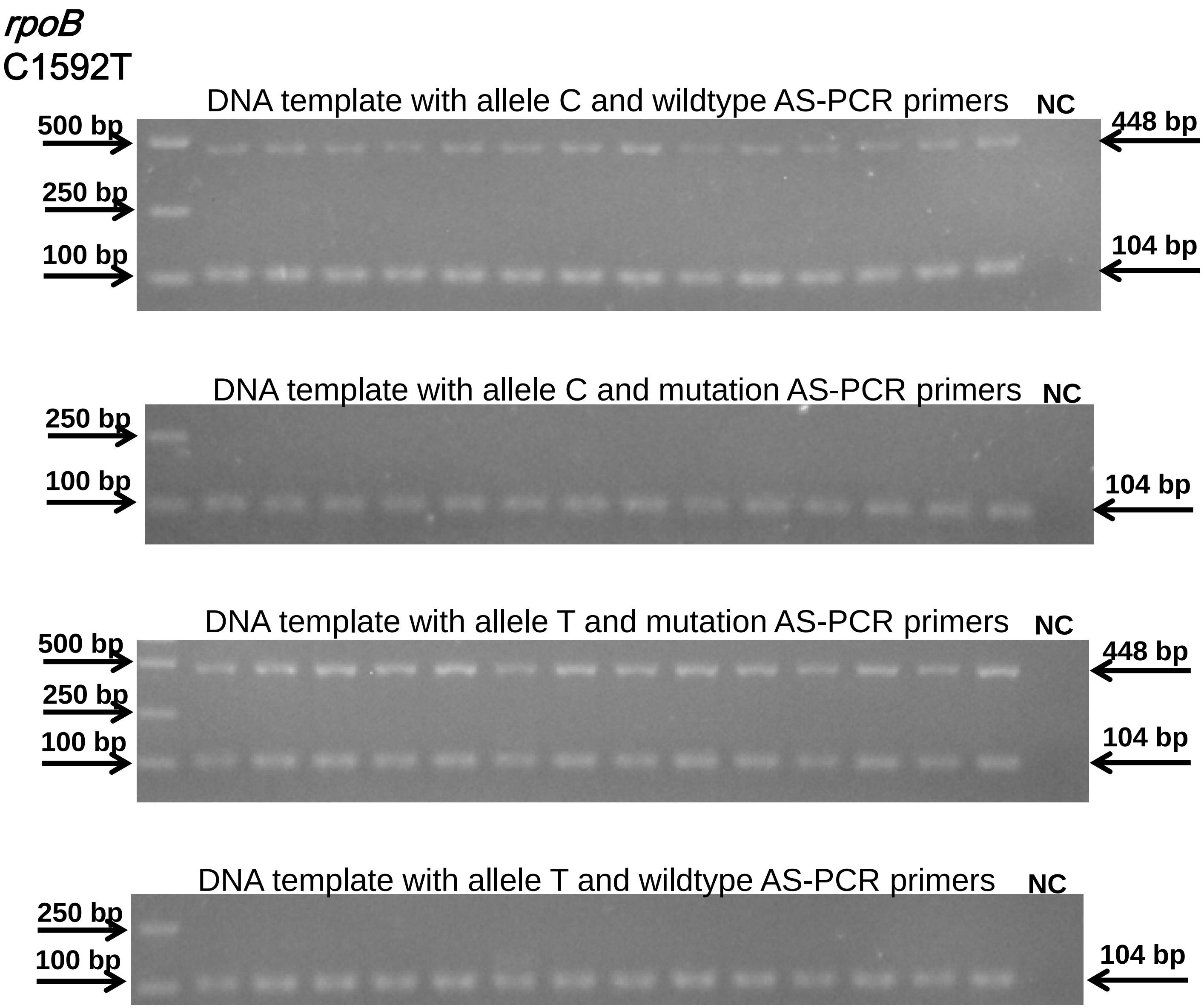

rpoB
C1592T
DNA template with allele C and wildtype AS-PCR primers
NC
500 bp
250 bp
100 bp
448 bp
104 bp
DNA template with allele C and mutation AS-PCR primers
NC
250 bp
100 bp
104 bp
DNA template with allele T and mutation AS-PCR primers
NC
500 bp
250 bp
100 bp
448 bp
104 bp
DNA template with allele T and wildtype AS-PCR primers
NC
250 bp
100 bp
104 bp

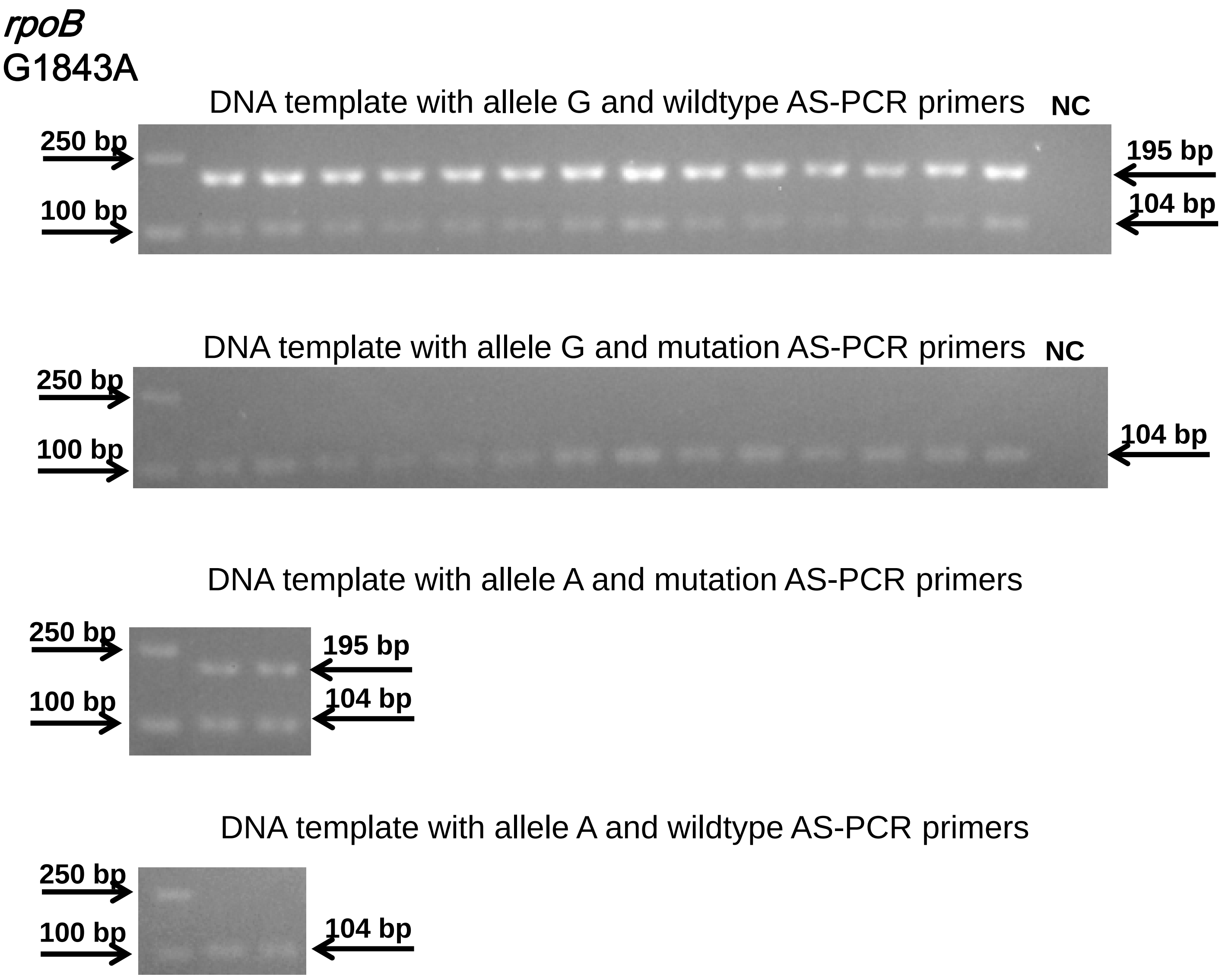
rpoB
G1843A
DNA template with allele G and wildtype AS-PCR primers
NC
250 bp
100 bp
195 bp
104 bp
DNA template with allele G and mutation AS-PCR primers
NC
250 bp
100 bp
104 bp
DNA template with allele A and mutation AS-PCR primers
250 bp
100 bp
195 bp
104 bp
DNA template with allele A and wildtype AS-PCR primers
250 bp
100 bp
104 bp

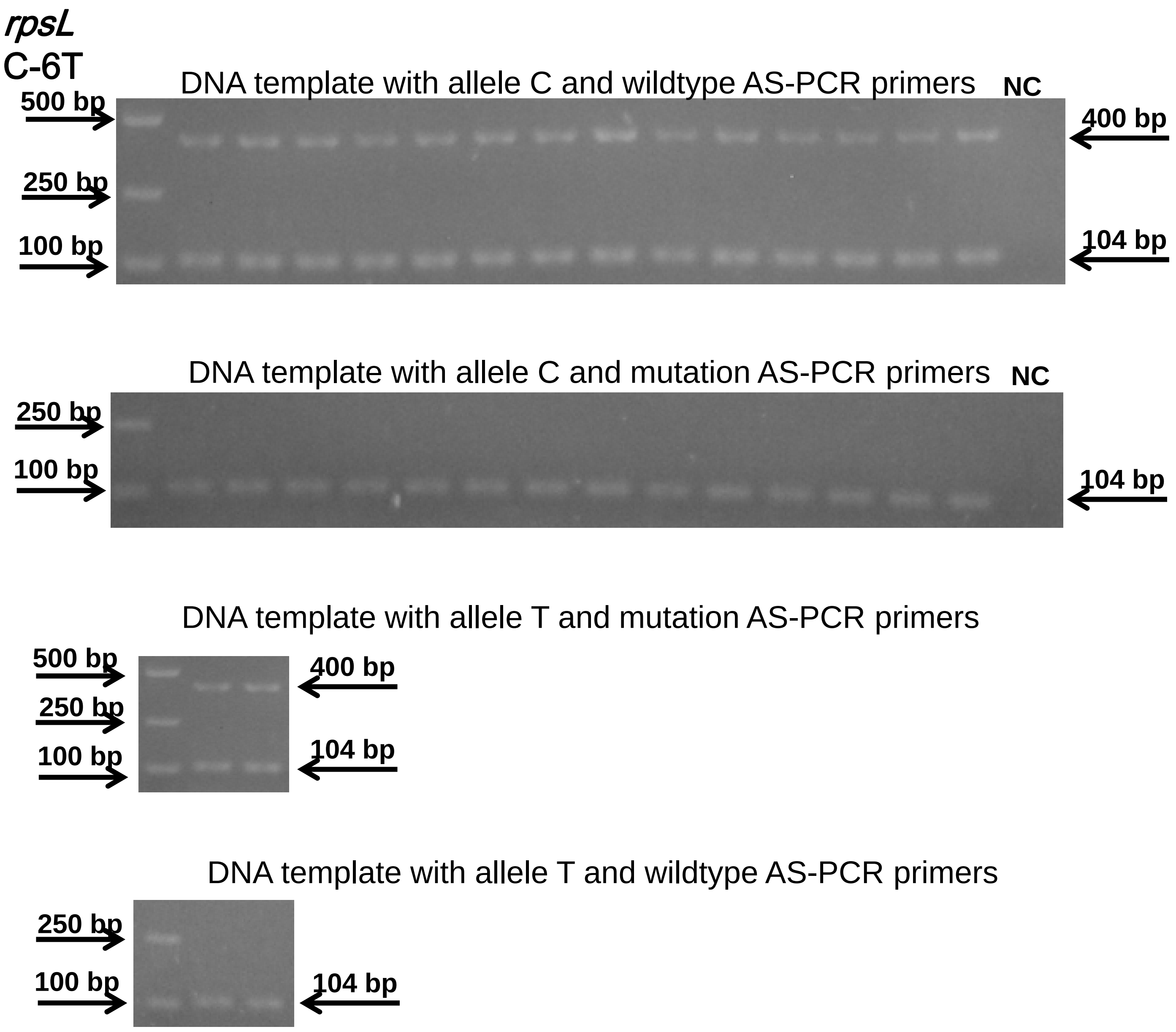
rpsL
C-6T
DNA template with allele C and wildtype AS-PCR primers
NC
500 bp
250 bp
100 bp
400 bp
104 bp
DNA template with allele C and mutation AS-PCR primers
NC
250 bp
100 bp
104 bp
DNA template with allele T and mutation AS-PCR primers
500 bp
250 bp
100 bp
400 bp
104 bp
DNA template with allele T and wildtype AS-PCR primers
250 bp
100 bp
104 bp

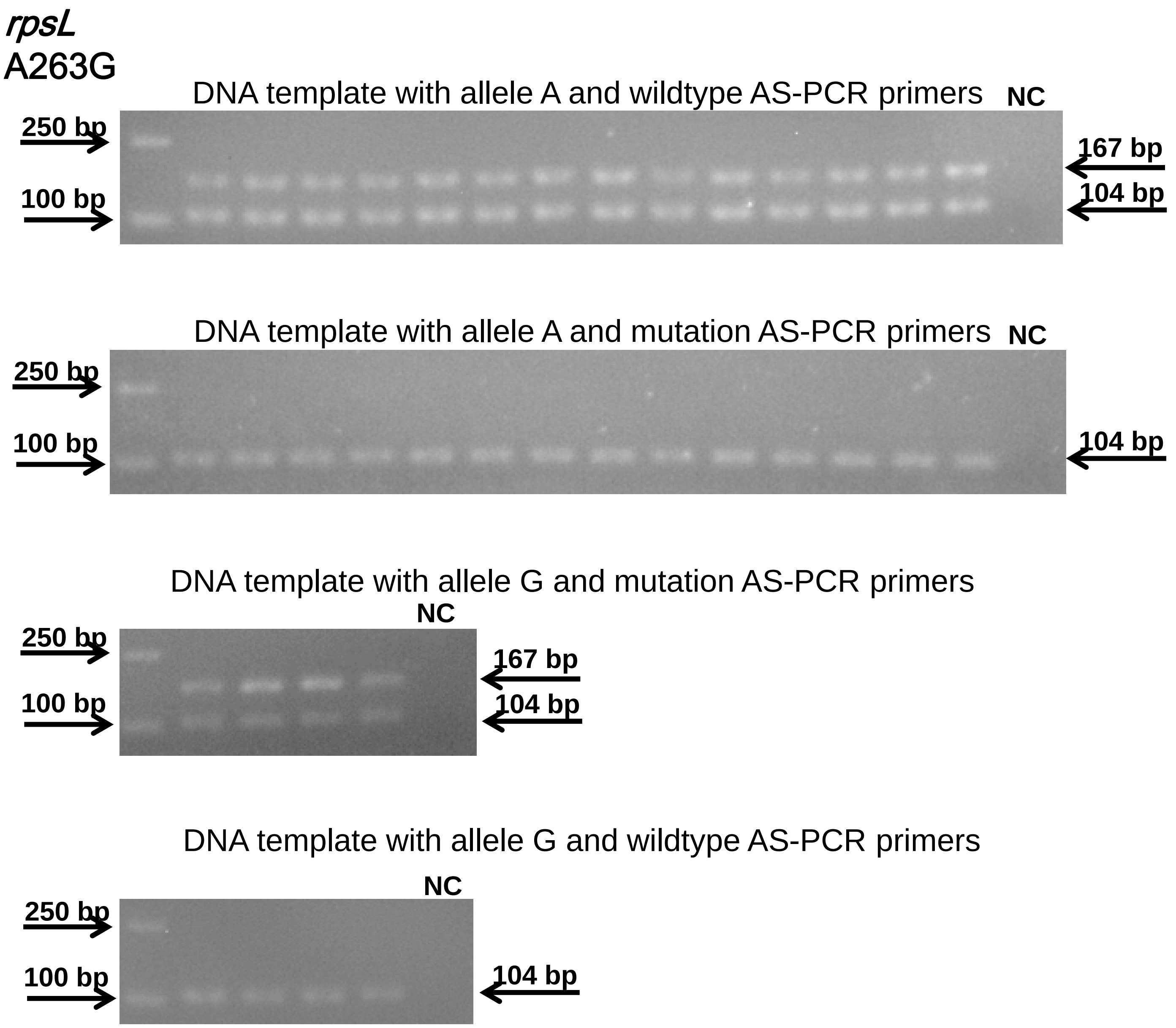
rpsL
A263G
DNA template with allele A and wildtype AS-PCR primers
NC
250 bp
100 bp
167 bp
104 bp
DNA template with allele A and mutation AS-PCR primers
NC
250 bp
100 bp
104 bp
DNA template with allele G and mutation AS-PCR primers
NC
250 bp
100 bp
167 bp
104 bp
DNA template with allele G and wildtype AS-PCR primers
NC
250 bp
100 bp
104 bp

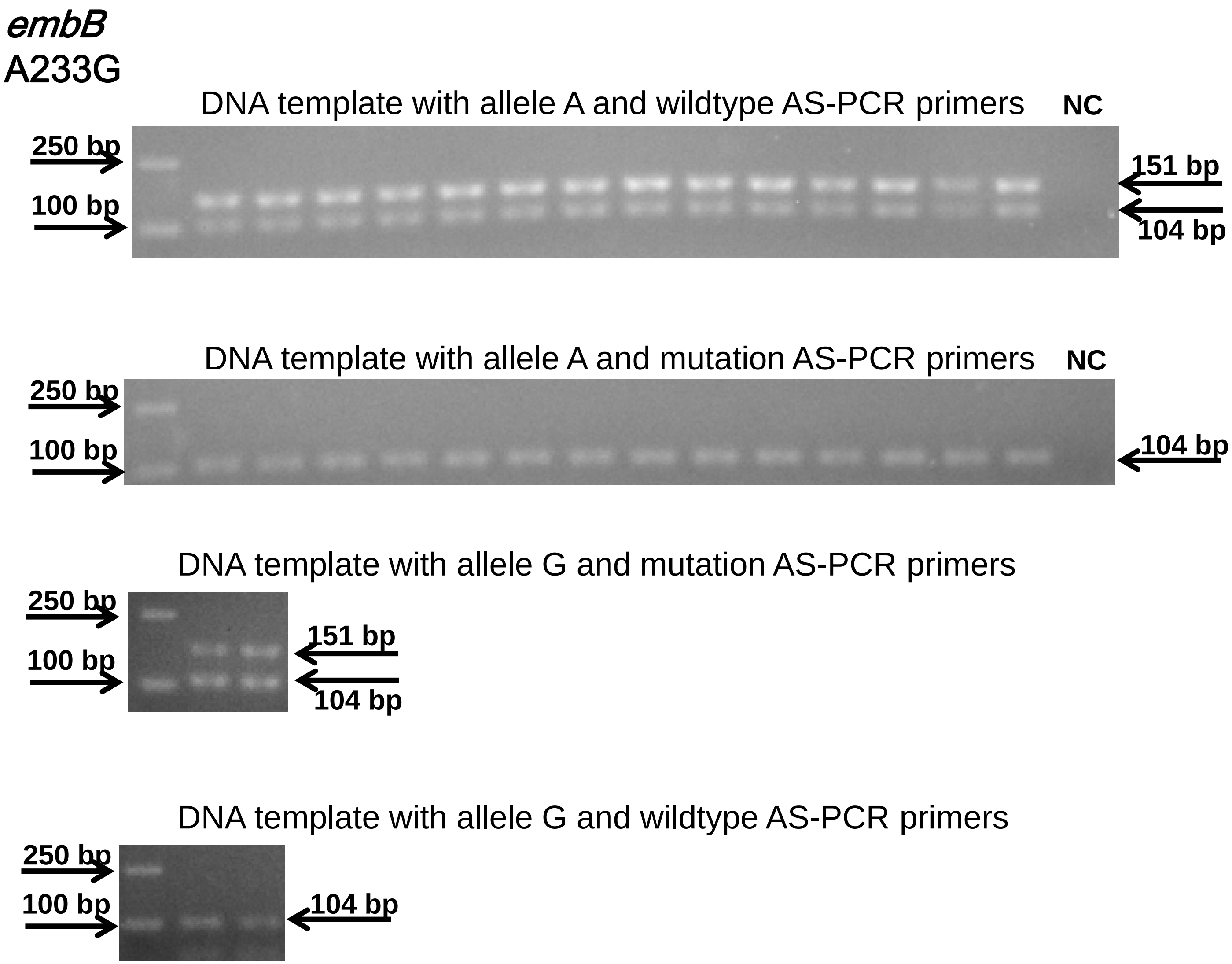
embB
A233G
DNA template with allele A and wildtype AS-PCR primers
NC
250 bp
100 bp
151 bp
104 bp
DNA template with allele A and mutation AS-PCR primers
NC
250 bp
100 bp
104 bp
DNA template with allele G and mutation AS-PCR primers
250 bp
100 bp
151 bp
104 bp
DNA template with allele G and wildtype AS-PCR primers
250 bp
100 bp
104 bp

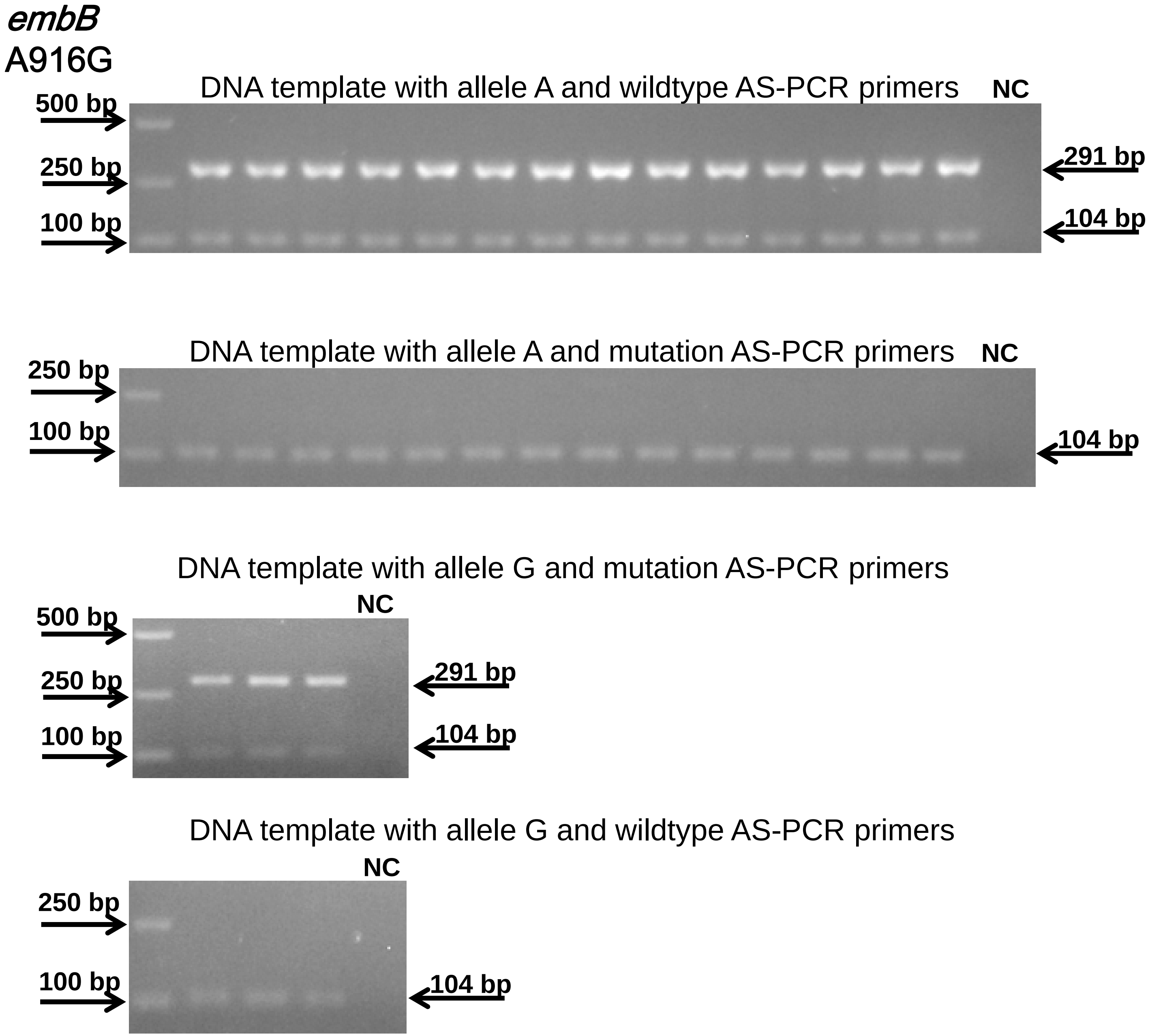

embB
A916G
DNA template with allele A and wildtype AS-PCR primers
NC
500 bp
250 bp
100 bp
291 bp
104 bp
DNA template with allele A and mutation AS-PCR primers
NC
250 bp
100 bp
104 bp
DNA template with allele G and mutation AS-PCR primers
NC
500 bp
250 bp
100 bp
291 bp
104 bp
DNA template with allele G and wildtype AS-PCR primers
NC
250 bp
100 bp
104 bp

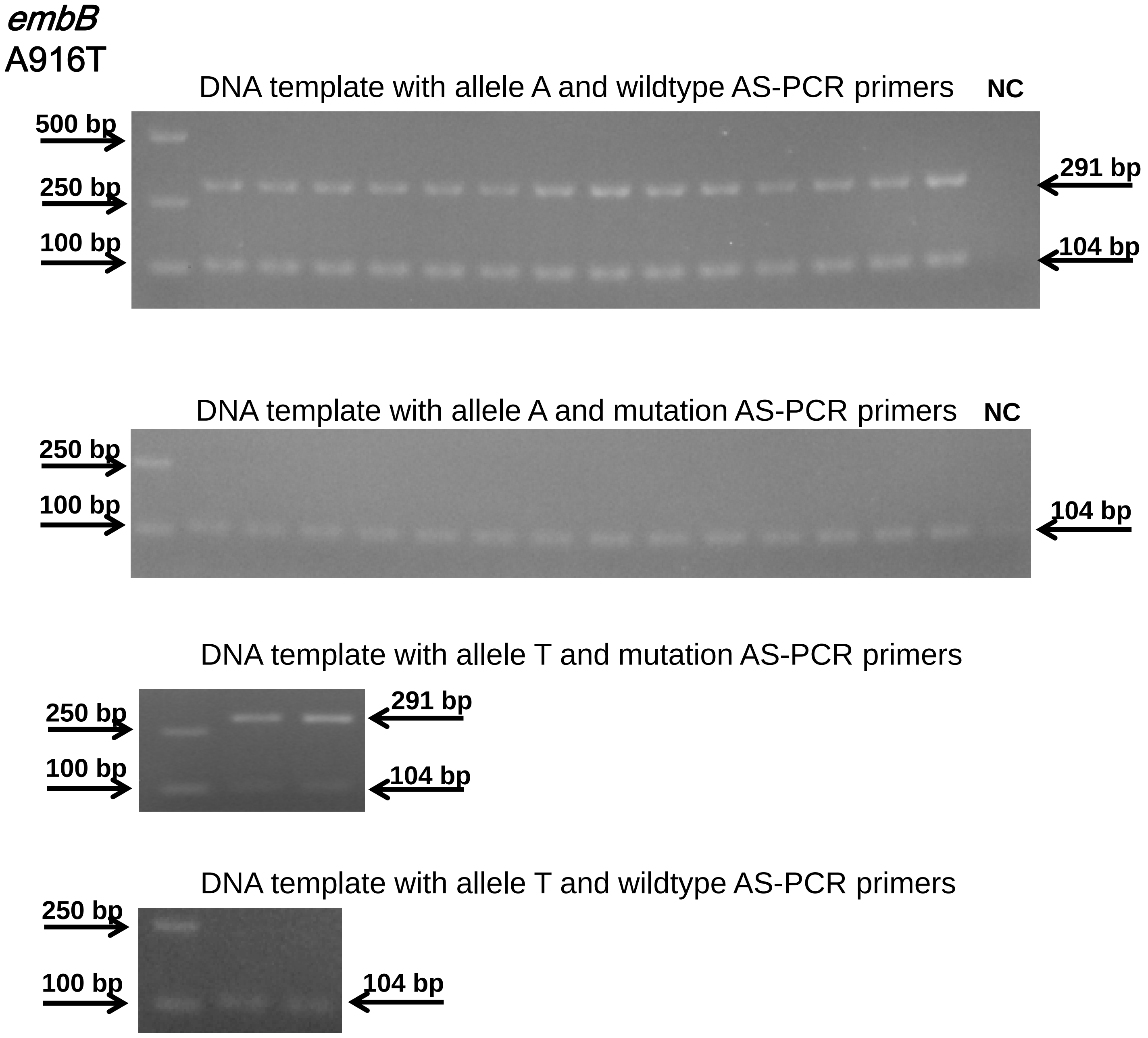
embB
A916T
DNA template with allele A and wildtype AS-PCR primers
NC
500 bp
250 bp
100 bp
291 bp
104 bp
DNA template with allele A and mutation AS-PCR primers
NC
250 bp
100 bp
104 bp
DNA template with allele T and mutation AS-PCR primers
250 bp
100 bp
291 bp
104 bp
DNA template with allele T and wildtype AS-PCR primers
250 bp
100 bp
104 bp

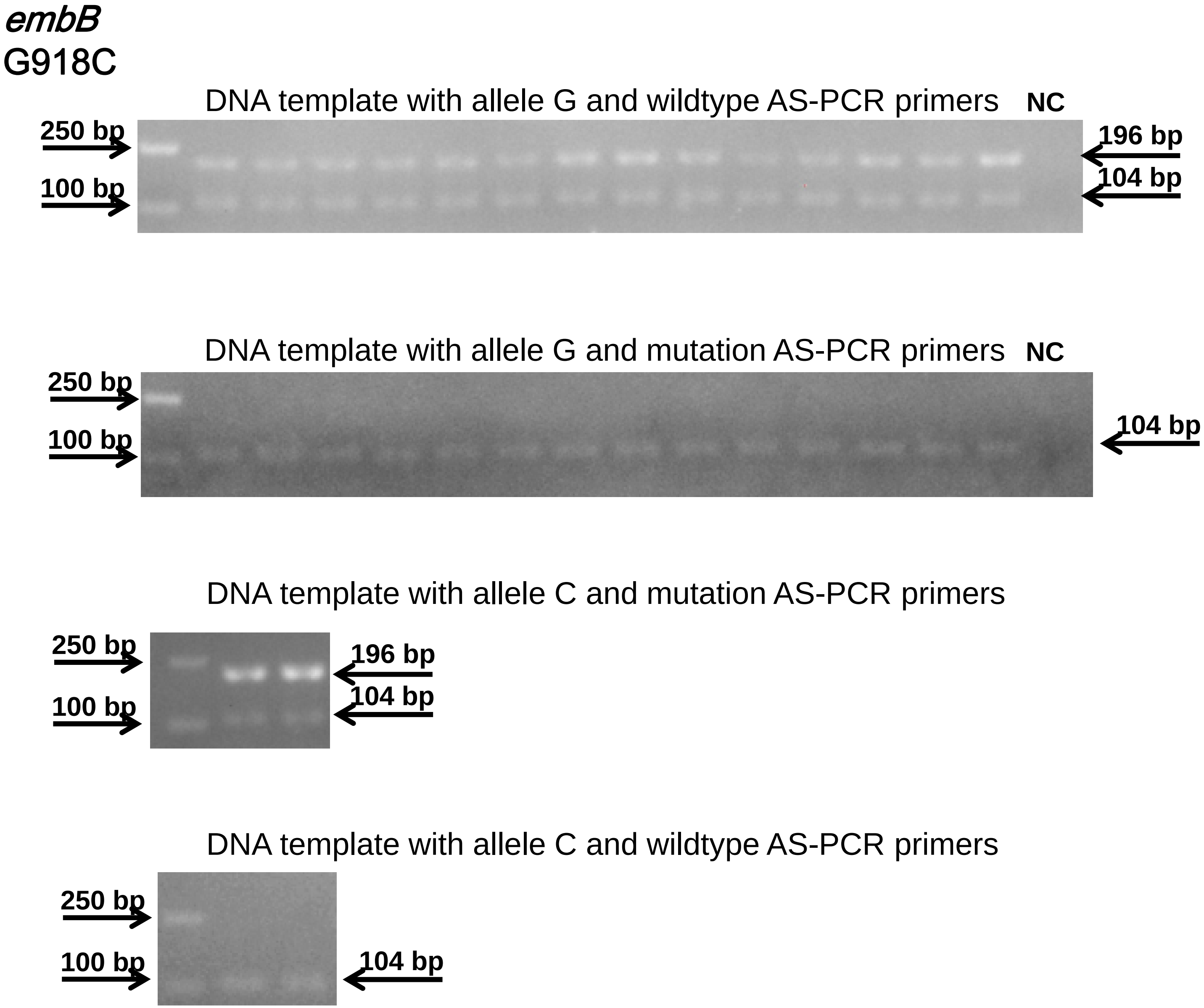
embB
G918C
DNA template with allele G and wildtype AS-PCR primers
NC
250 bp
100 bp
196 bp
104 bp
DNA template with allele G and mutation AS-PCR primers
NC
250 bp
100 bp
104 bp
DNA template with allele C and mutation AS-PCR primers
250 bp
100 bp
196 bp
104 bp
DNA template with allele C and wildtype AS-PCR primers
250 bp
100 bp
104 bp

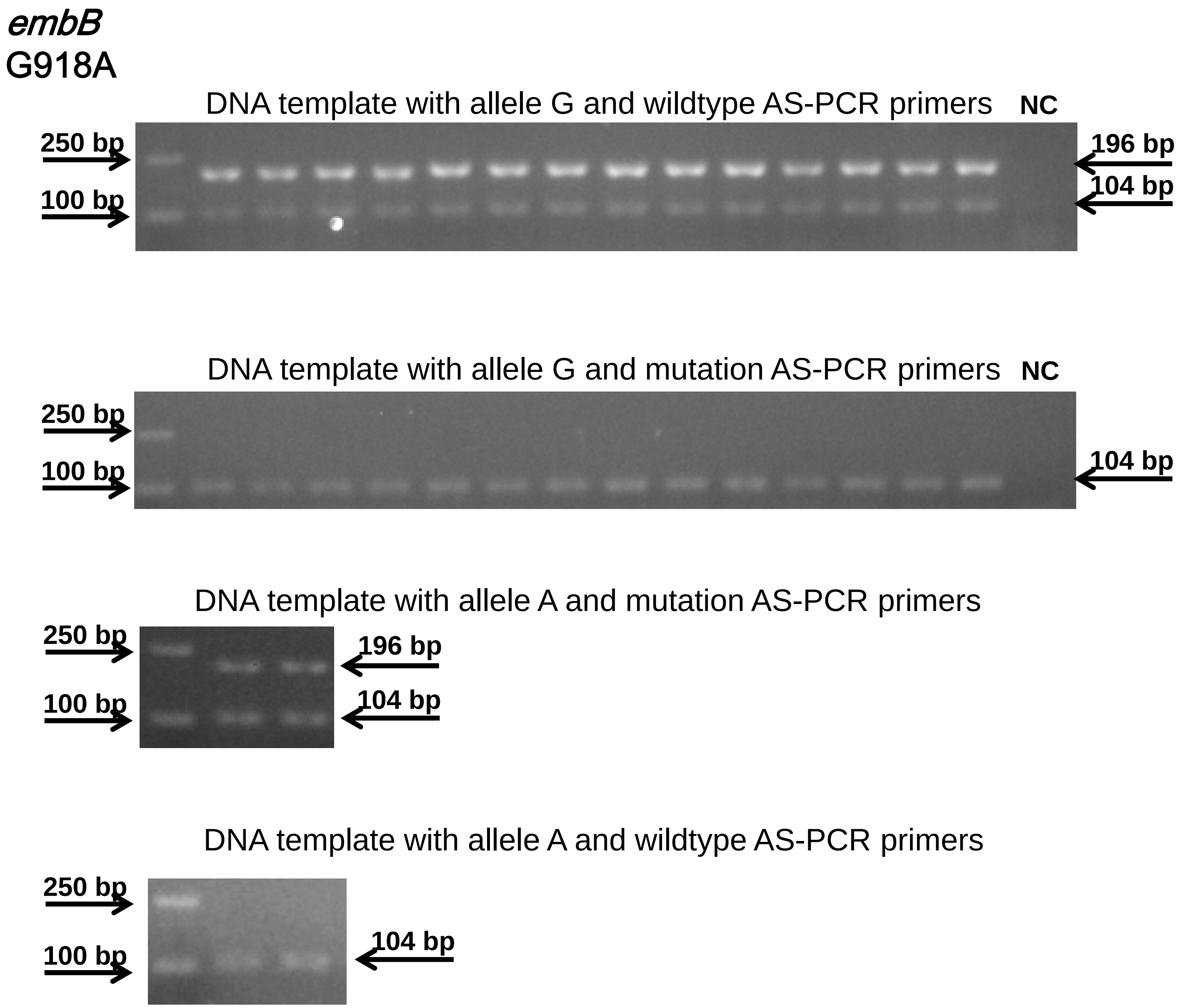
embB
G918A
DNA template with allele G and wildtype AS-PCR primers
NC
250 bp
100 bp
196 bp
104 bp
DNA template with allele G and mutation AS-PCR primers
NC
250 bp
100 bp
104 bp
DNA template with allele A and mutation AS-PCR primers
250 bp
100 bp
196 bp
104 bp
DNA template with allele A and wildtype AS-PCR primers
250 bp
100 bp
104 bp

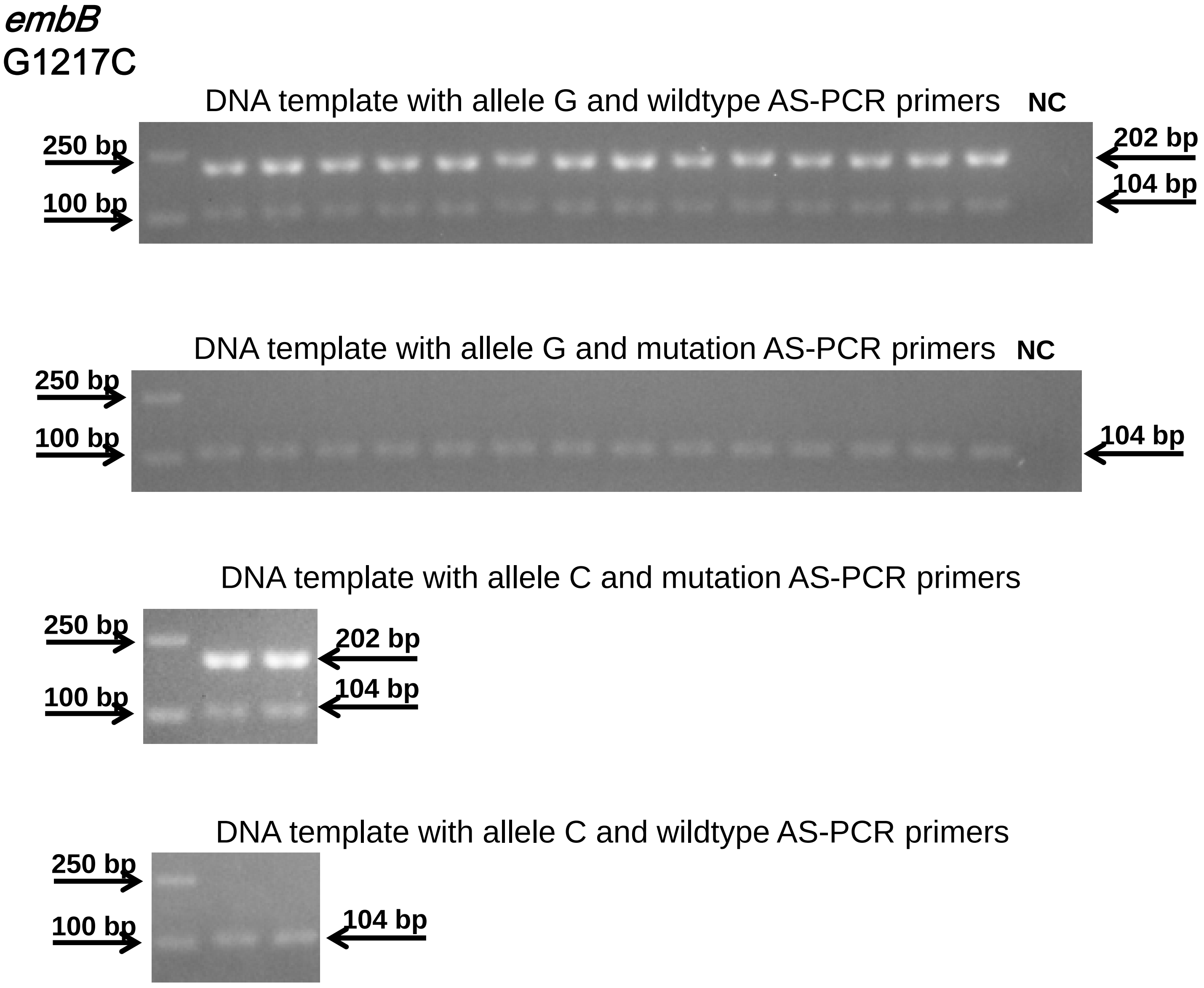

embB
G1217C
DNA template with allele G and wildtype AS-PCR primers
NC
250 bp
100 bp
202 bp
104 bp
DNA template with allele G and mutation AS-PCR primers
NC
250 bp
100 bp
104 bp
DNA template with allele C and mutation AS-PCR primers
250 bp
100 bp
202 bp
104 bp
DNA template with allele C and wildtype AS-PCR primers
250 bp
100 bp
104 bp
