# Supplementary figures and images for: Rapid screening mutations of first-line-drug-resistant genes in Mycobacterium tuberculosis strains by allele-specific real-time quantitative PCR
Source: PeerJ. 2019 Apr 1;7:e6696. doi: 10.7717/peerj.6696 (PMC6448557; doi:10.7717/peerj.6696)

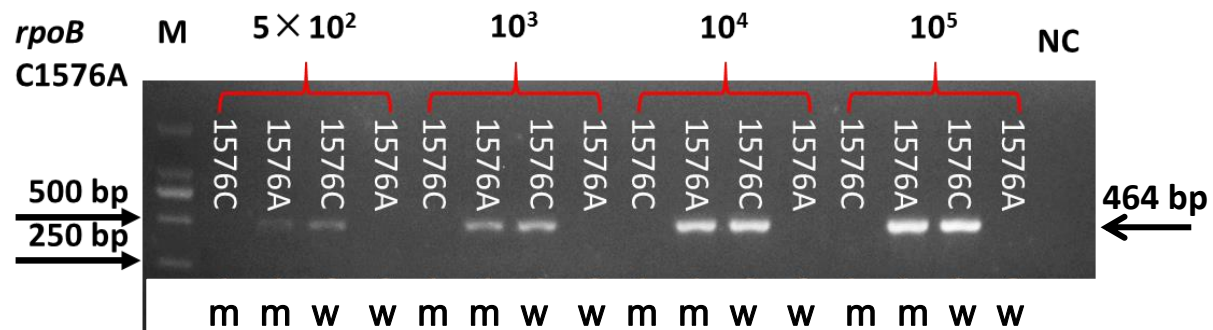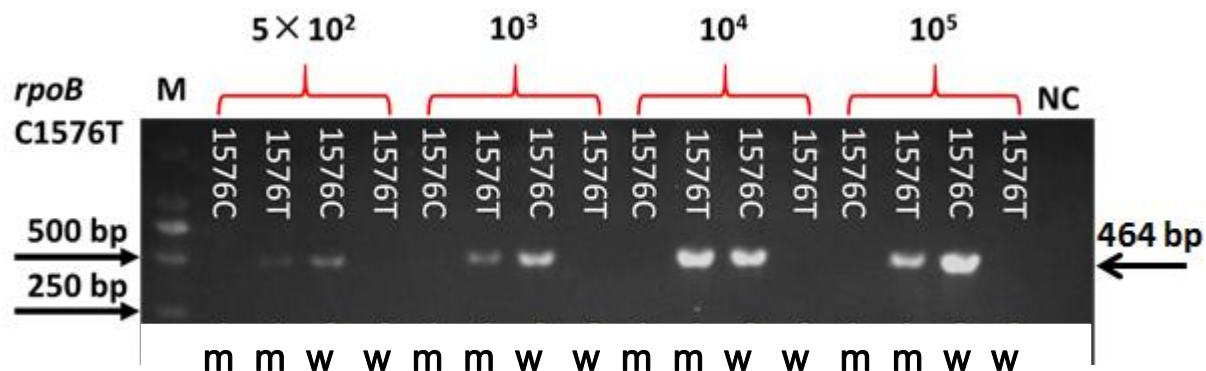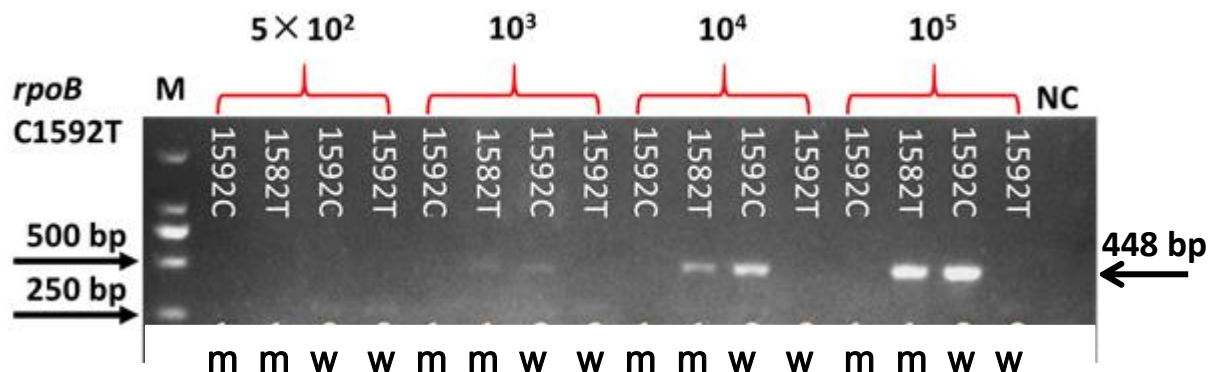

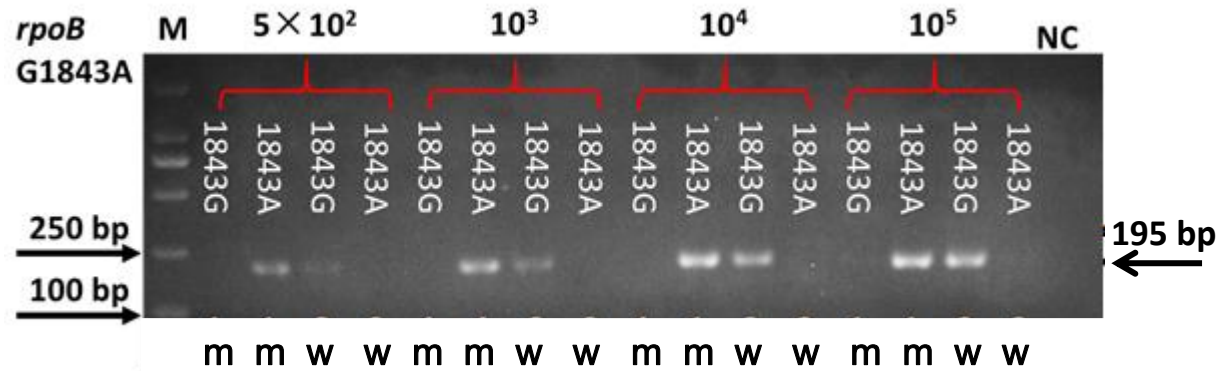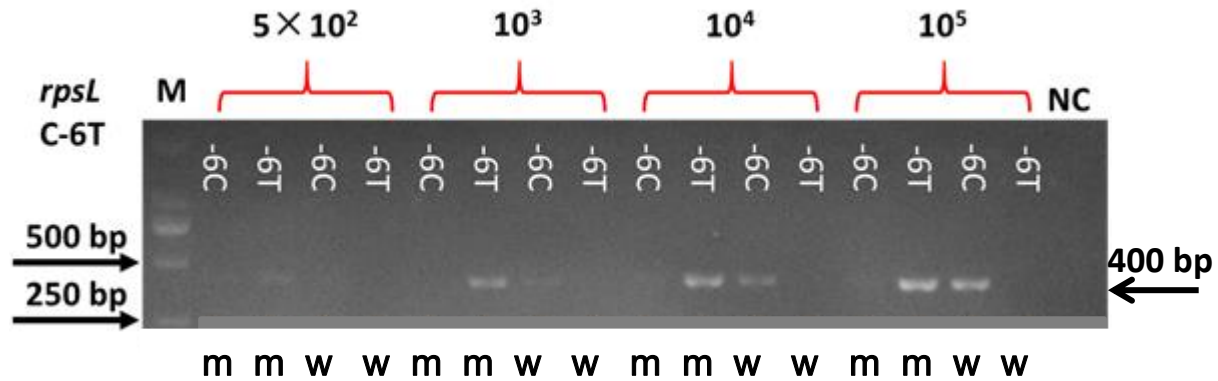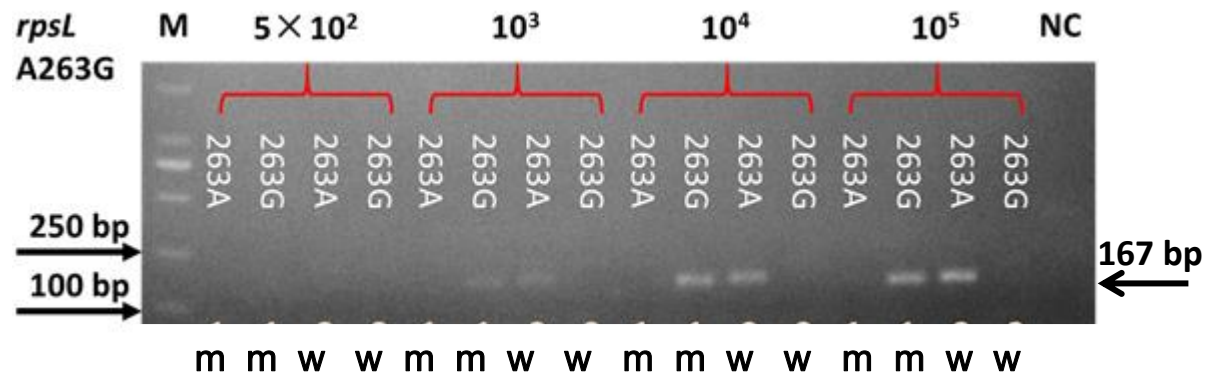

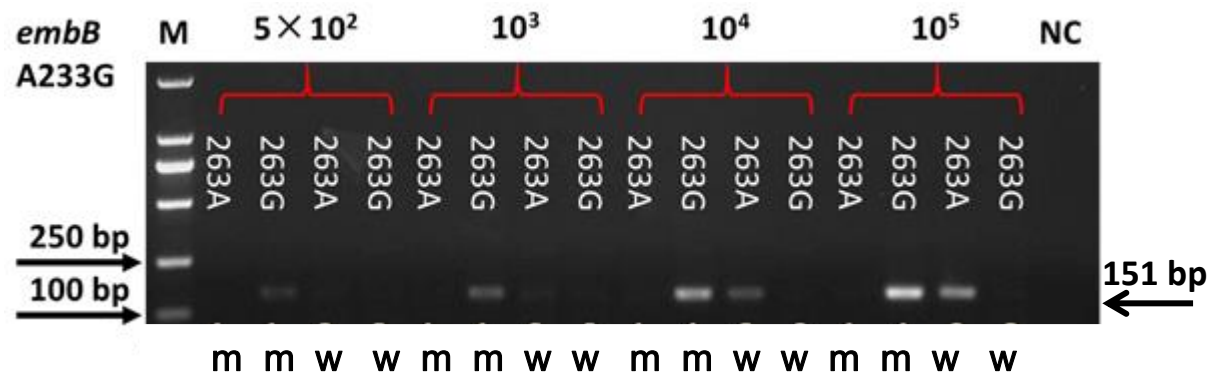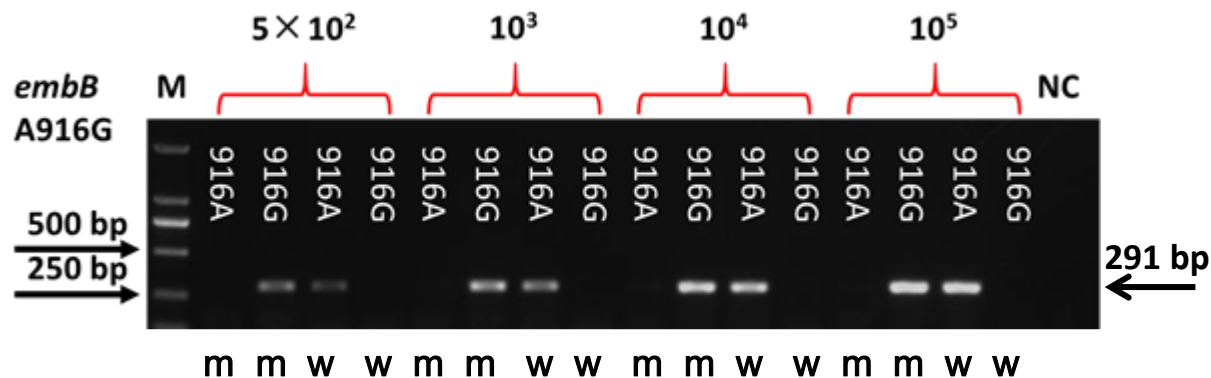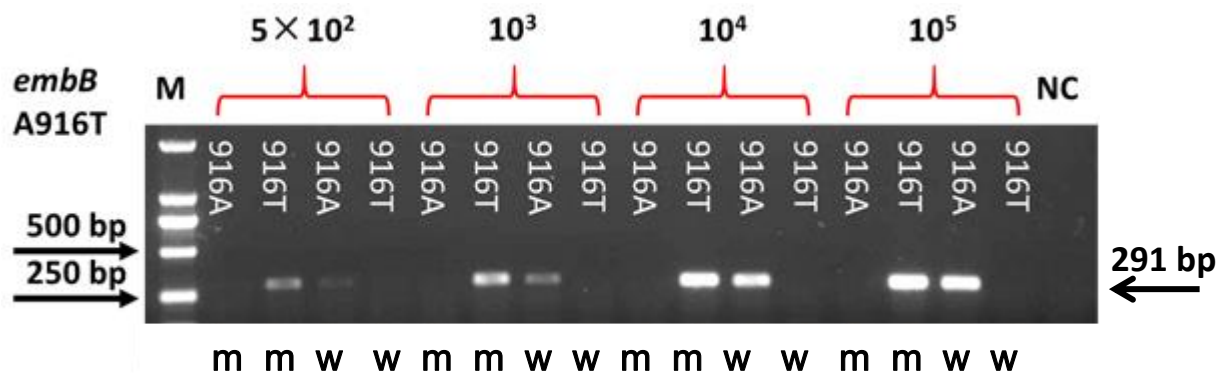

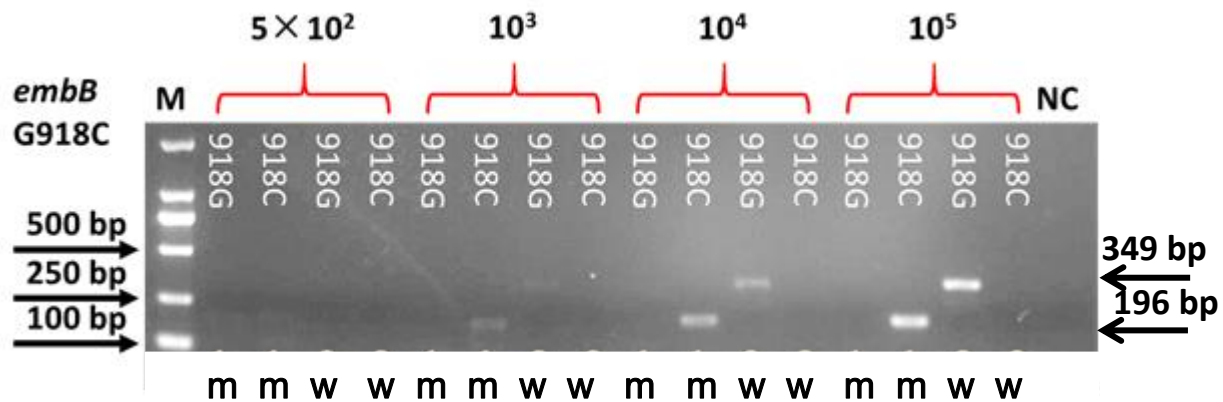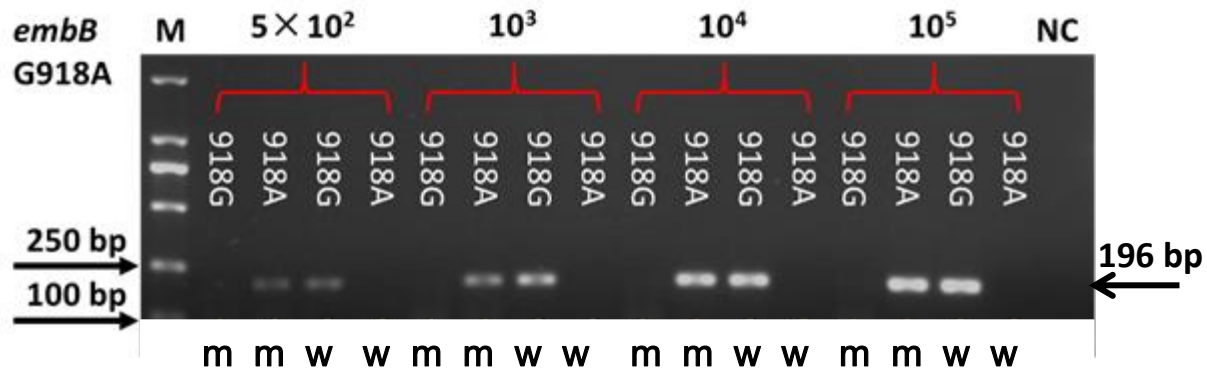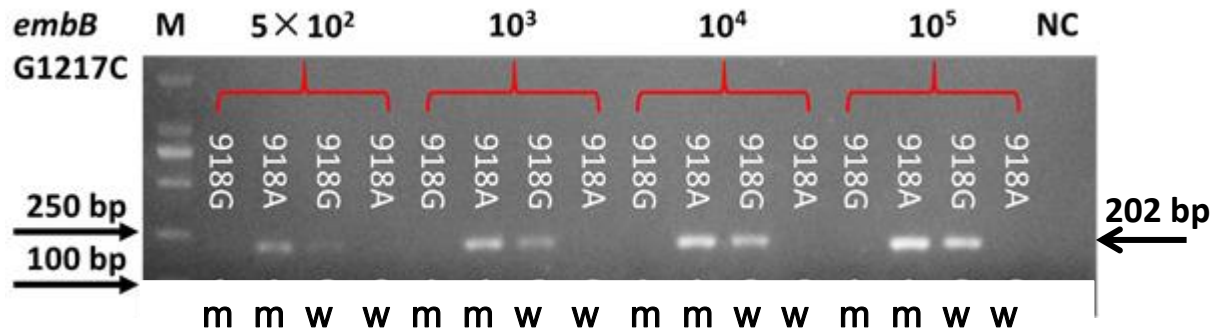

Supplement: Supplemental Information 2 [file peerj-07-6696-s002.pdf]
