# Supplementary material for: Rapid screening mutations of first-line-drug-resistant genes in Mycobacterium tuberculosis strains by allele-specific real-time quantitative PCR
Source: PeerJ. 2019 Apr 1;7:e6696. doi: 10.7717/peerj.6696 (PMC6448557; doi:10.7717/peerj.6696)
Supplement: Supplemental Information 3 [file peerj-07-6696-s003.pdf]

katG 898T

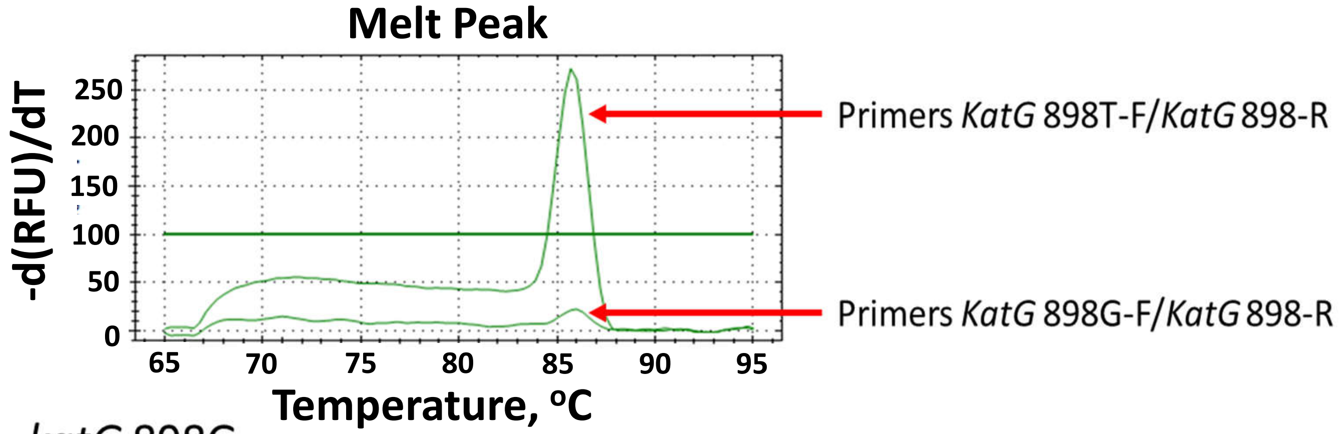

katG 898G

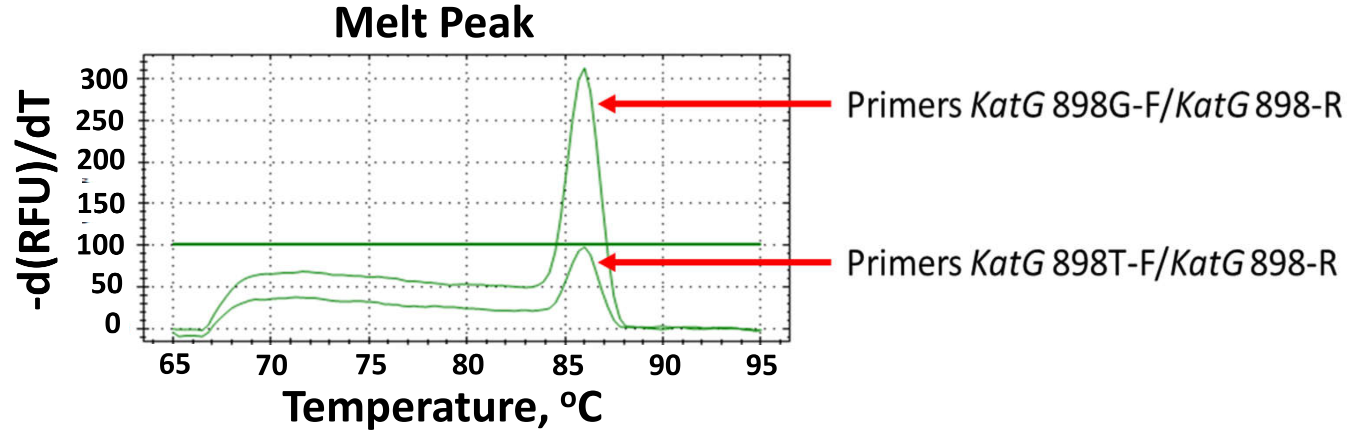

Melt Peak

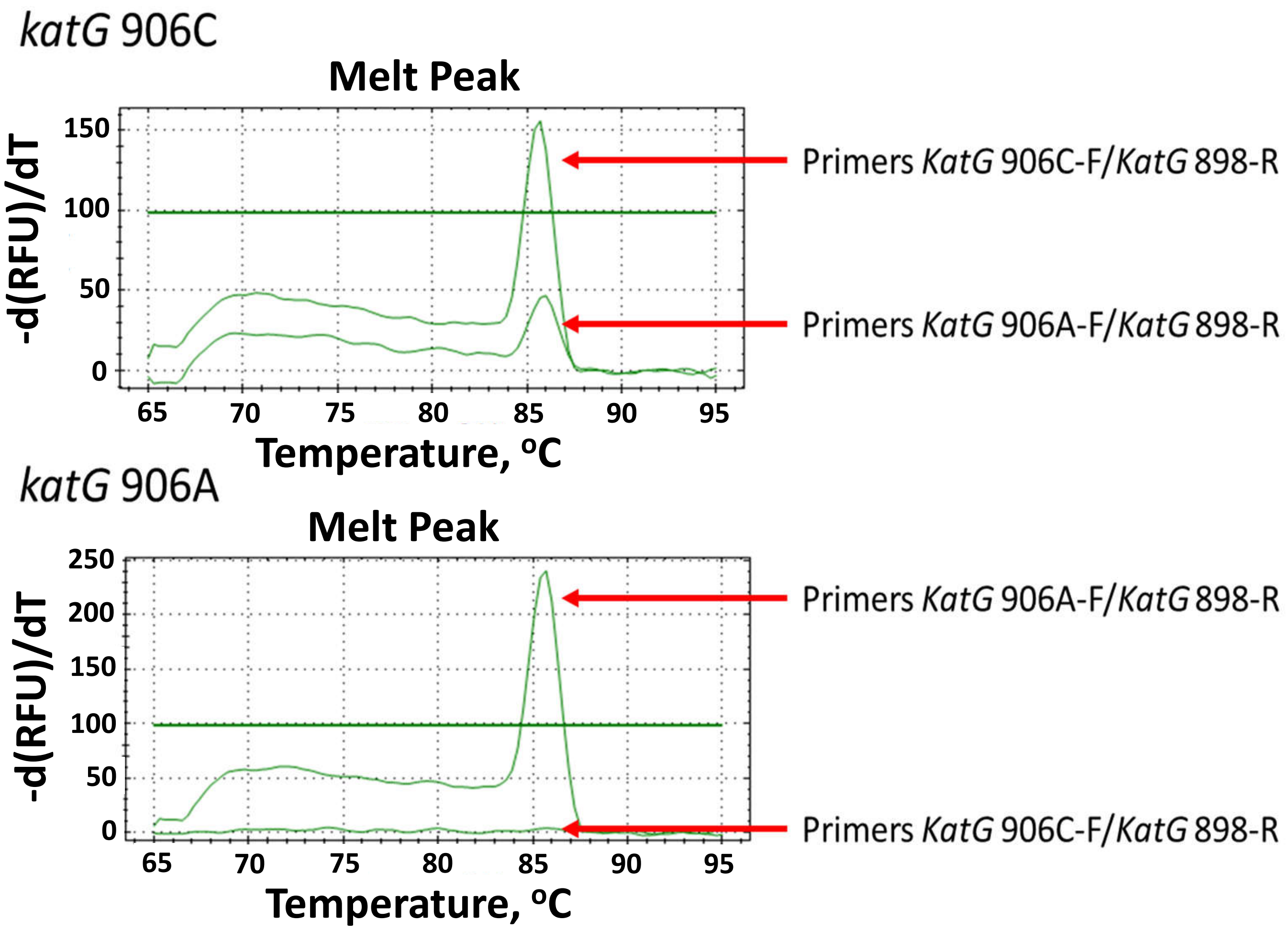
katG 906C
Melt Peak
-d(RFU)/dT
150
100
50
0
65
70
75
80
85
90
95
Temperature, °C
Primers KatG 906C-F/KatG 898-R
Primers KatG 906A-F/KatG 898-R
katG 906A
Melt Peak
-d(RFU)/dT
250
200
150
100
50
0
65
70
75
80
85
90
95
Temperature, °C
Primers KatG 906A-F/KatG 898-R
Primers KatG 906C-F/KatG 898-R

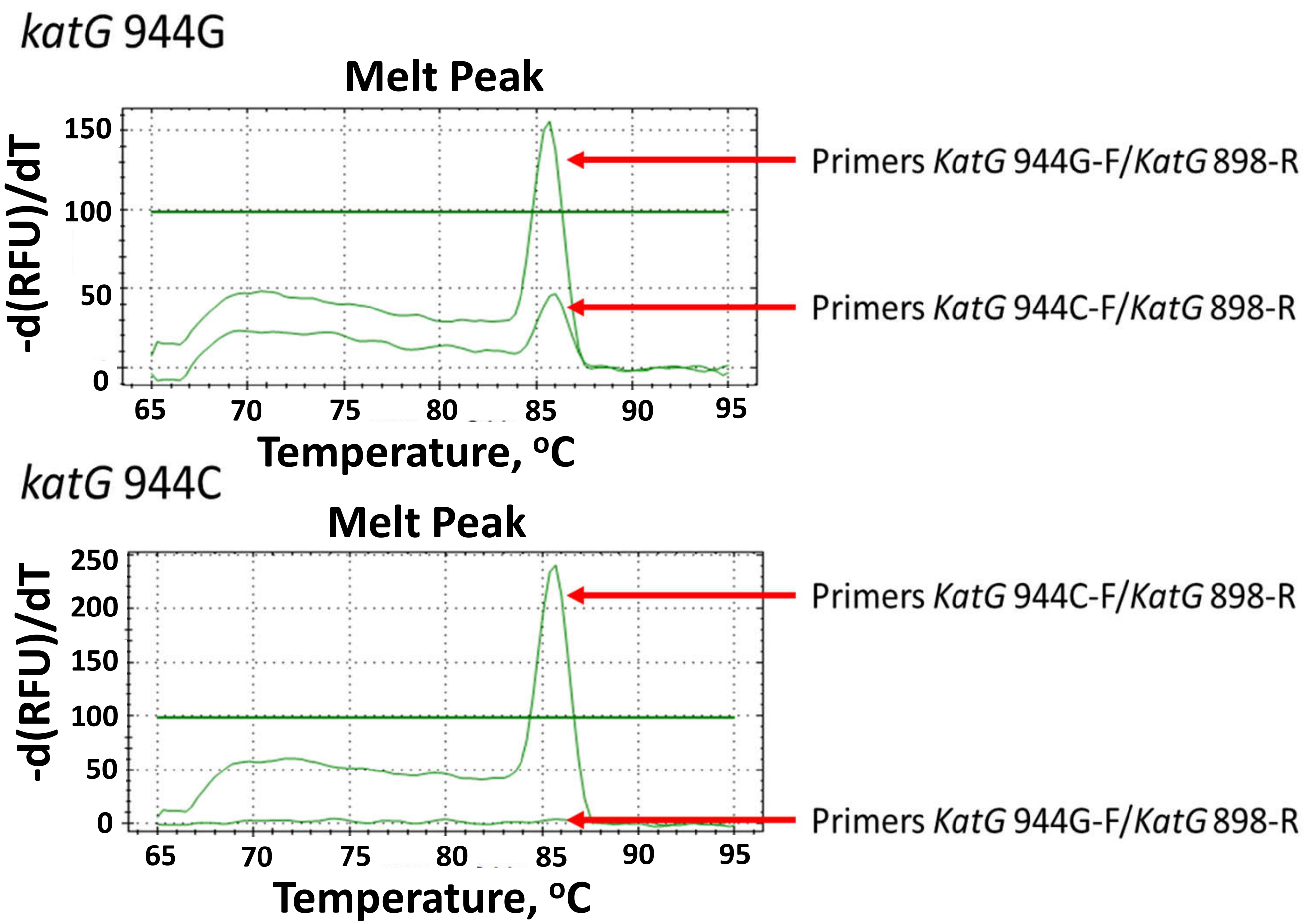

katG 944G
Melt Peak
-d(RFU)/dT
150
100
50
0
65
70
75
80
85
90
95
Temperature, °C
Primers KatG 944G-F/KatG 898-R
Primers KatG 944C-F/KatG 898-R
katG 944C
Melt Peak
-d(RFU)/dT
250
200
150
100
50
0
65
70
75
80
85
90
95
Temperature, °C
Primers KatG 944C-F/KatG 898-R
Primers KatG 944G-F/KatG 898-R

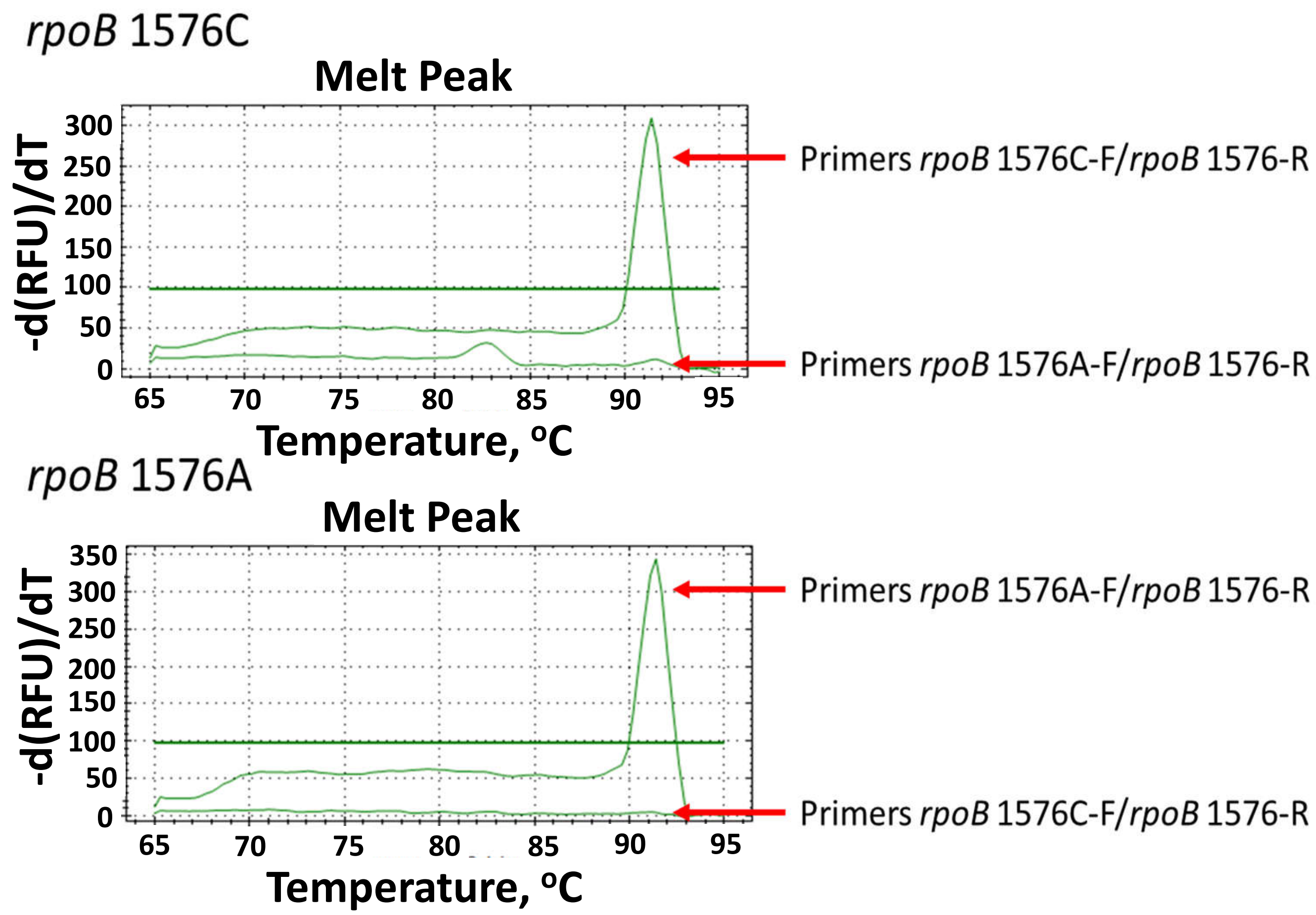

rpoB 1576C
Melt Peak
-d(RFU)/dT
300
250
200
150
100
50
0
65
70
75
80
85
90
95
Temperature, °C
Primers rpoB 1576C-F/rpoB 1576-R
Primers rpoB 1576A-F/rpoB 1576-R
rpoB 1576A
Melt Peak
-d(RFU)/dT
350
300
250
200
150
100
50
0
65
70
75
80
85
90
95
Temperature, °C
Primers rpoB 1576A-F/rpoB 1576-R
Primers rpoB 1576C-F/rpoB 1576-R

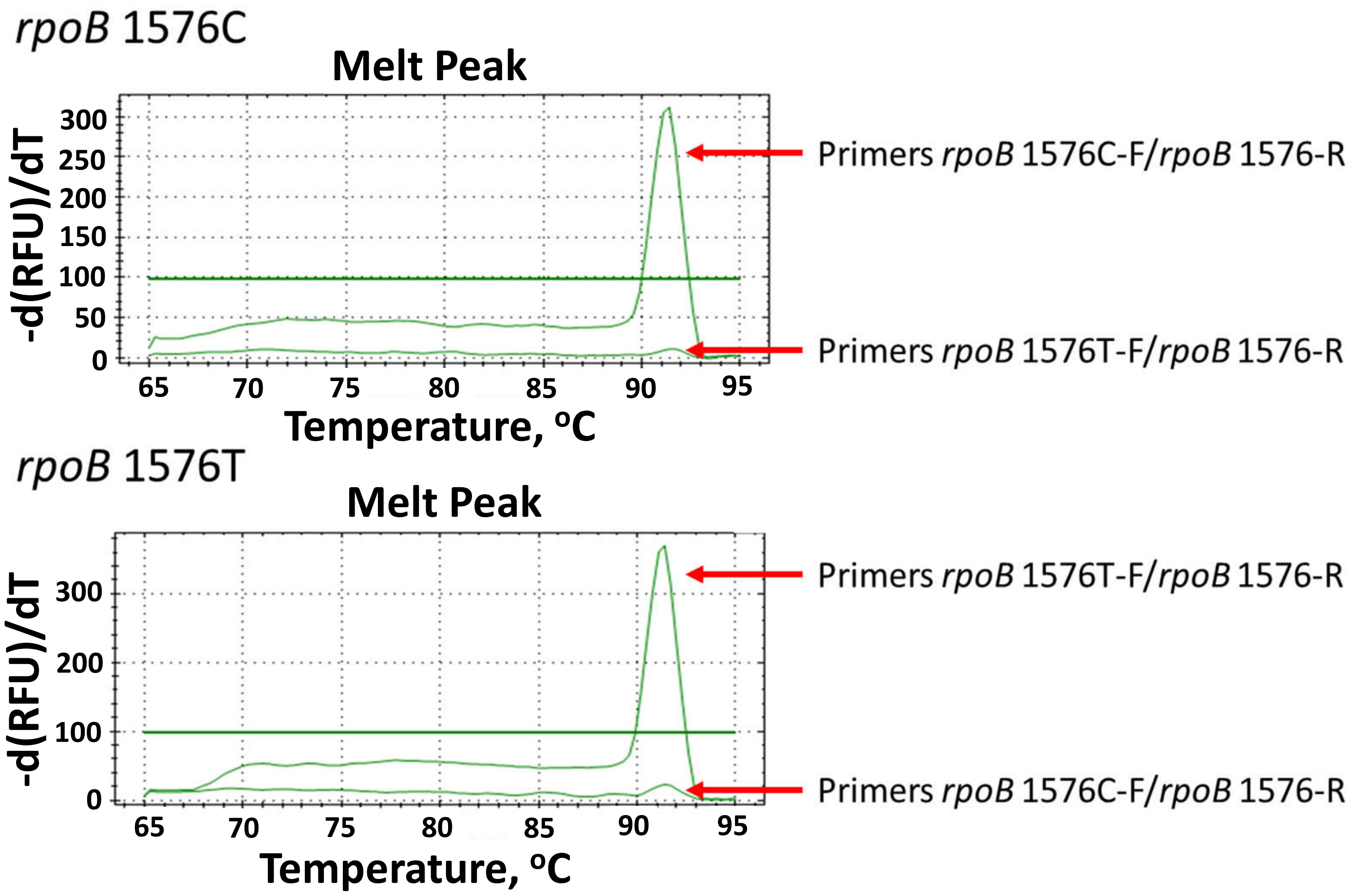

rpoB 1576C
Melt Peak
-d(RFU)/dT
300
250
200
150
100
50
0
65
70
75
80
85
90
95
Temperature, °C
Primers rpoB 1576C-F/rpoB 1576-R
Primers rpoB 1576T-F/rpoB 1576-R
rpoB 1576T
Melt Peak
-d(RFU)/dT
300
200
100
0
65
70
75
80
85
90
95
Temperature, °C
Primers rpoB 1576T-F/rpoB 1576-R
Primers rpoB 1576C-F/rpoB 1576-R

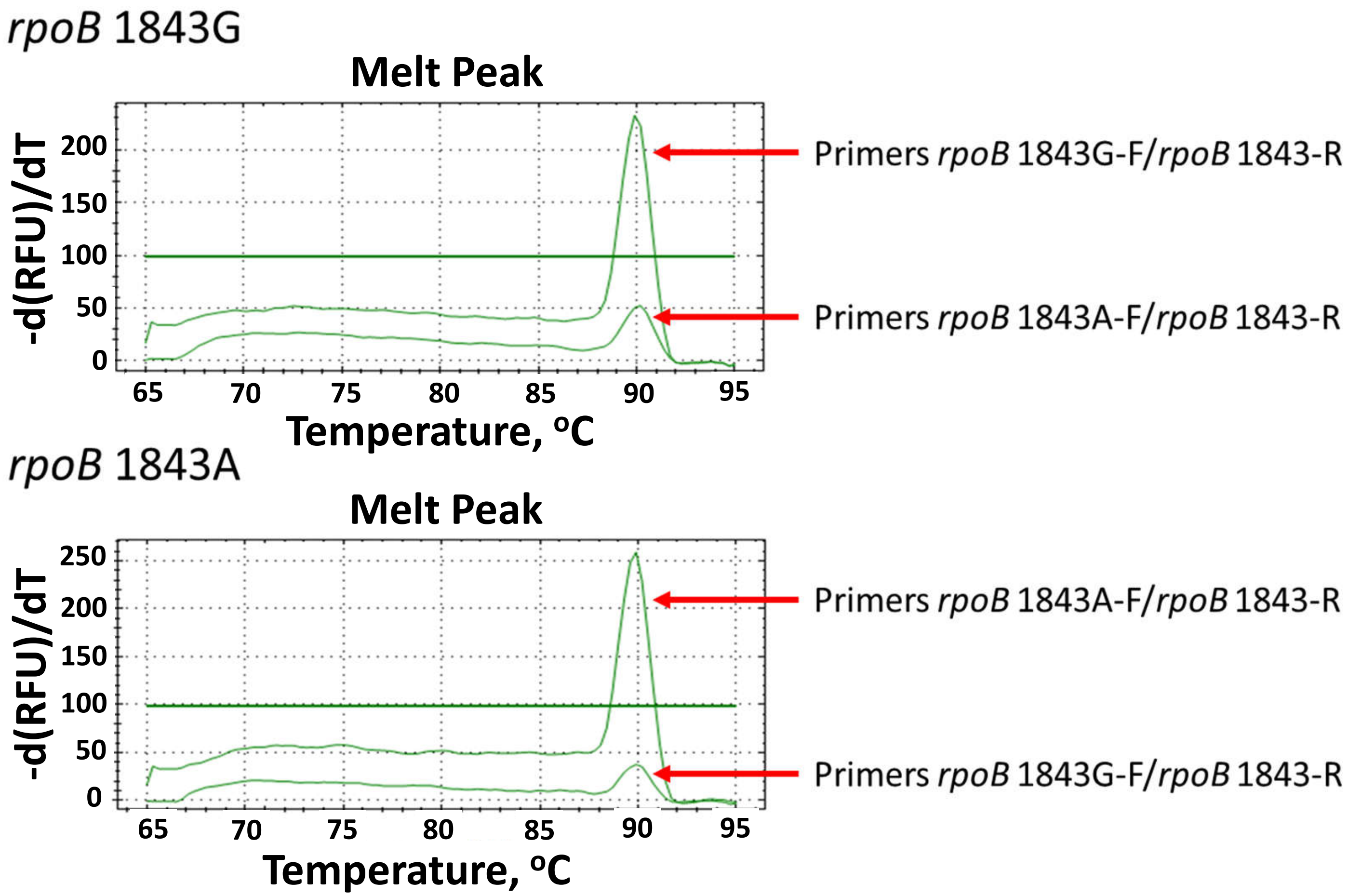
rpoB 1843G
Melt Peak
-d(RFU)/dT
0
50
100
150
200
65
70
75
80
85
90
95
Temperature, °C
Primers *rpoB* 1843G-F/*rpoB* 1843-R
Primers *rpoB* 1843A-F/*rpoB* 1843-R
rpoB 1843A
Melt Peak
-d(RFU)/dT
0
50
100
150
200
250
65
70
75
80
85
90
95
Temperature, °C
Primers *rpoB* 1843A-F/*rpoB* 1843-R
Primers *rpoB* 1843G-F/*rpoB* 1843-R

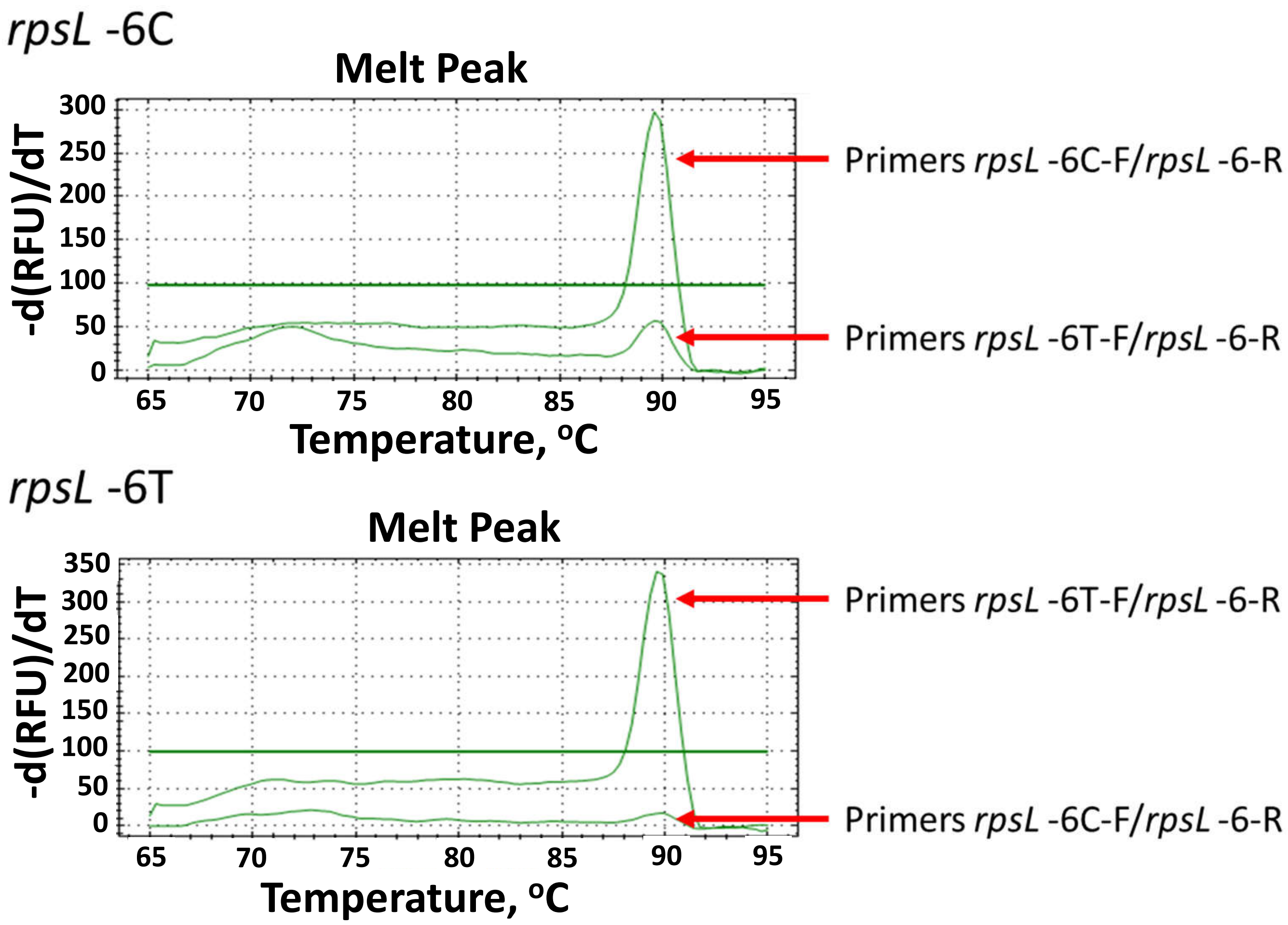
rpsL -6C
Melt Peak
-d(RFU)/dT
300
250
200
150
100
50
0
65
70
75
80
85
90
95
Temperature, °C
Primers rpsL -6C-F/rpsL -6-R
Primers rpsL -6T-F/rpsL -6-R
rpsL -6T
Melt Peak
-d(RFU)/dT
350
300
250
200
150
100
50
0
65
70
75
80
85
90
95
Temperature, °C
Primers rpsL -6T-F/rpsL -6-R
Primers rpsL -6C-F/rpsL -6-R

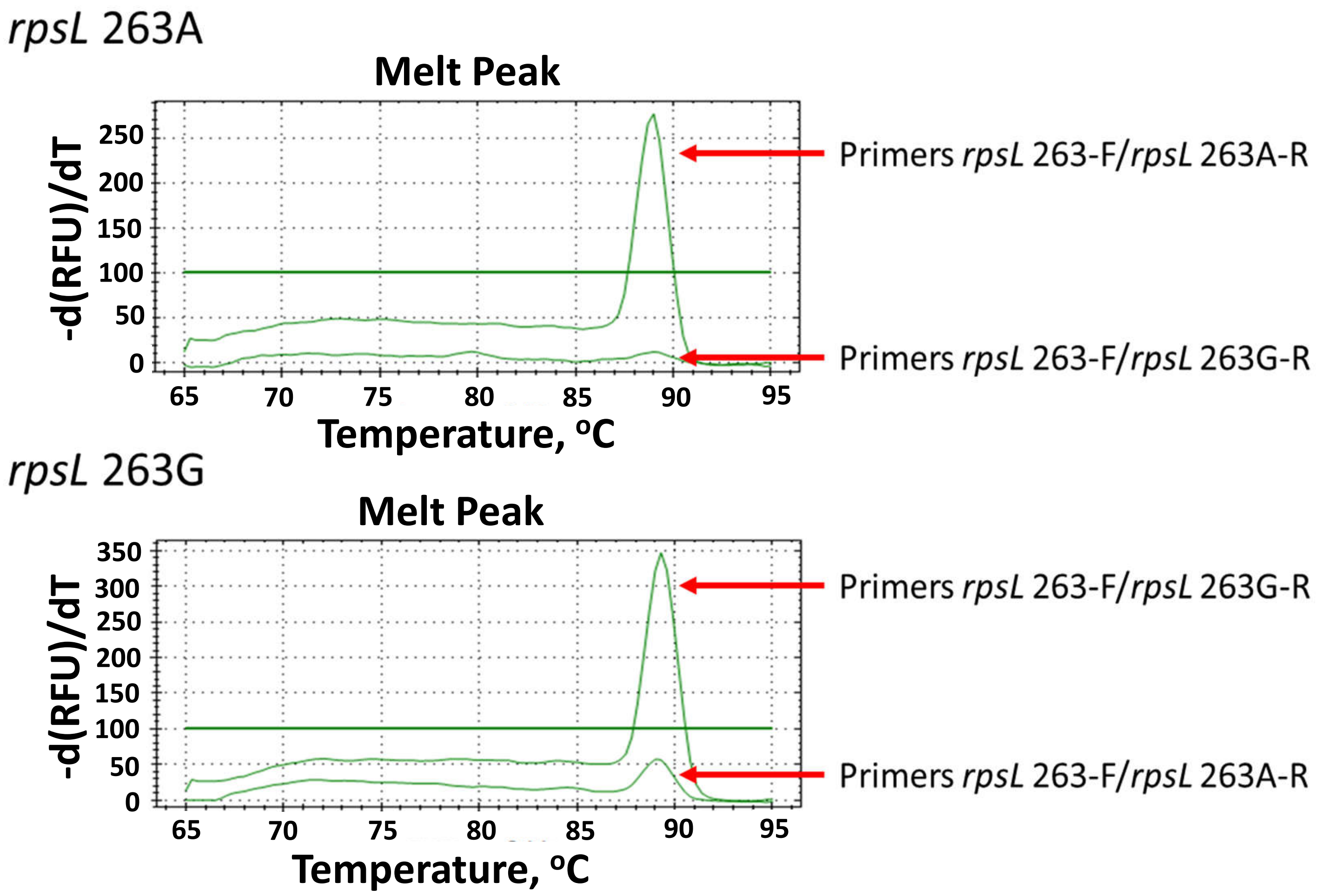
rpsL 263A
Melt Peak
-d(RFU)/dT
250
200
150
100
50
0
65
70
75
80
85
90
95
Temperature, ºC
Primers rpsL 263-F/rpsL 263A-R
Primers rpsL 263-F/rpsL 263G-R
rpsL 263G
Melt Peak
-d(RFU)/dT
350
300
250
200
150
100
50
0
65
70
75
80
85
90
95
Temperature, ºC
Primers rpsL 263-F/rpsL 263G-R
Primers rpsL 263-F/rpsL 263A-R

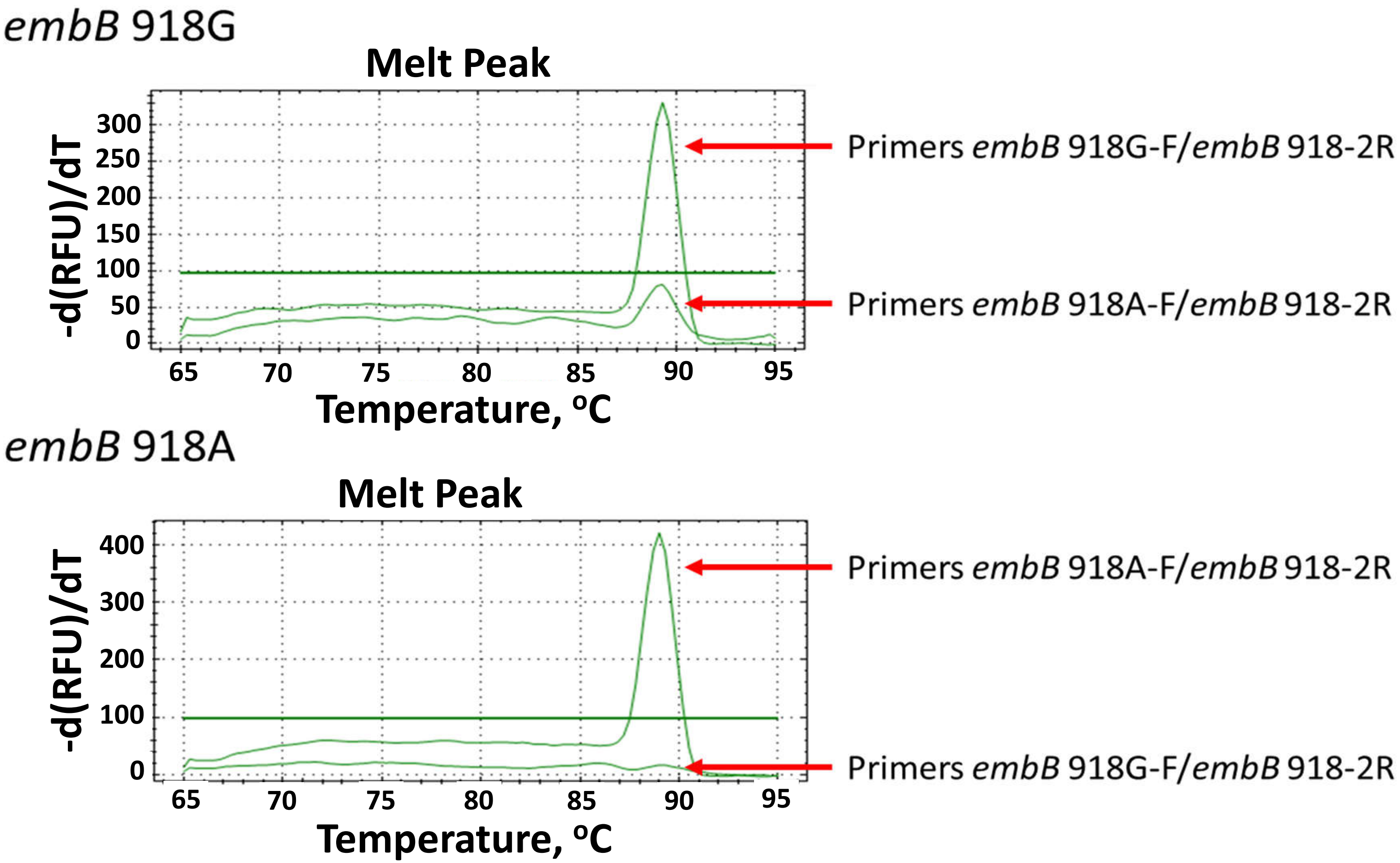
embB 918G
Melt Peak
-d(RFU)/dT
300
250
200
150
100
50
0
65
70
75
80
85
90
95
Temperature, °C
Primers embB 918G-F/embB 918-2R
Primers embB 918A-F/embB 918-2R
embB 918A
Melt Peak
-d(RFU)/dT
400
300
200
100
0
65
70
75
80
85
90
95
Temperature, °C
Primers embB 918A-F/embB 918-2R
Primers embB 918G-F/embB 918-2R

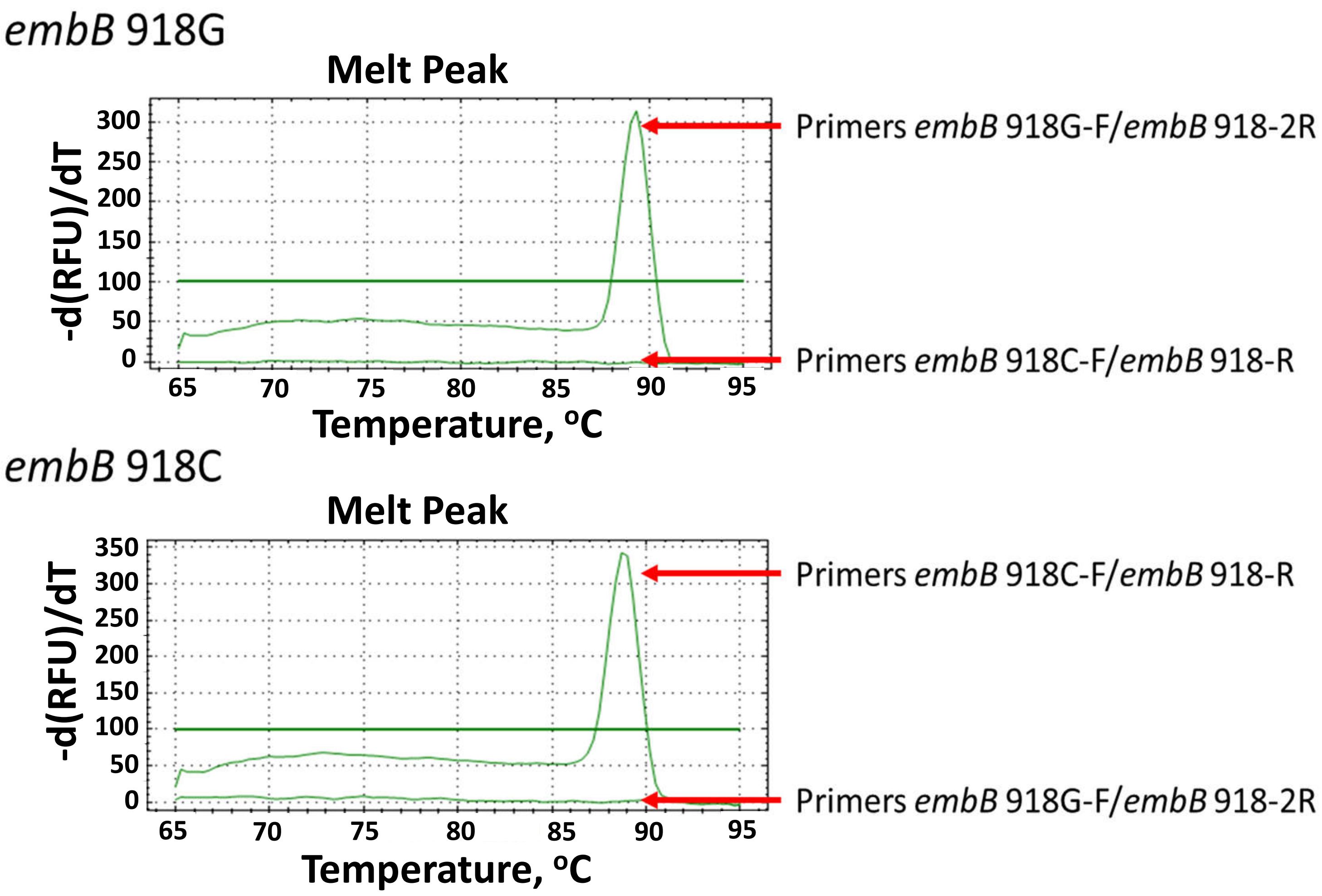
embB 918G
Melt Peak
-d(RFU)/dT
300
250
200
150
100
50
0
65
70
75
80
85
90
95
Temperature, °C
Primers *embB* 918G-F/*embB* 918-2R
Primers *embB* 918C-F/*embB* 918-R
embB 918C
Melt Peak
-d(RFU)/dT
350
300
250
200
150
100
50
0
65
70
75
80
85
90
95
Temperature, °C
Primers *embB* 918C-F/*embB* 918-R
Primers *embB* 918G-F/*embB* 918-2R

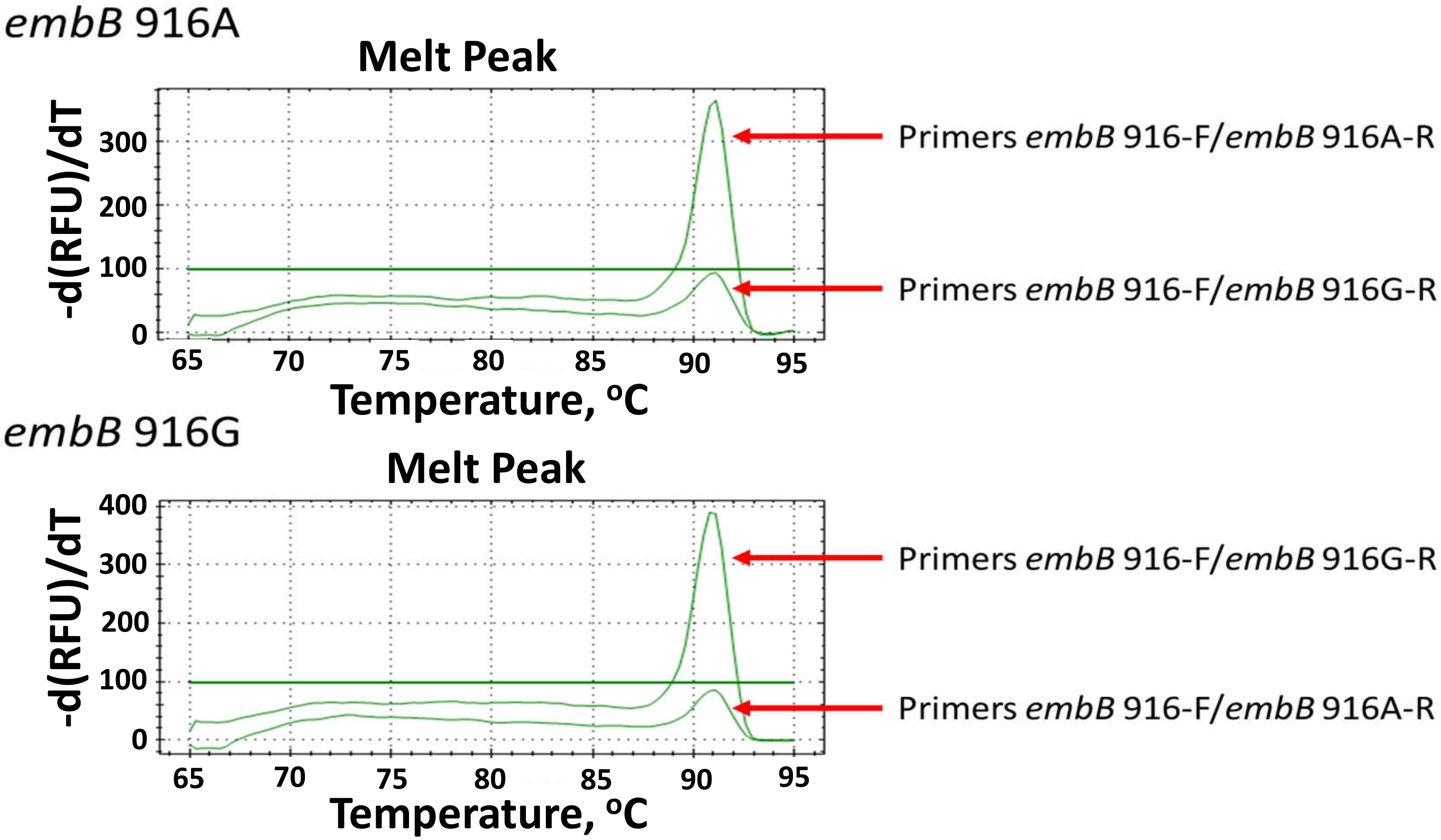
embB 916A
Melt Peak
-d(RFU)/dT
0
100
200
300
65
70
75
80
85
90
95
Temperature, °C
Primers embB 916-F/embB 916A-R
Primers embB 916-F/embB 916G-R
embB 916G
Melt Peak
-d(RFU)/dT
0
100
200
300
400
65
70
75
80
85
90
95
Temperature, °C
Primers embB 916-F/embB 916G-R
Primers embB 916-F/embB 916A-R

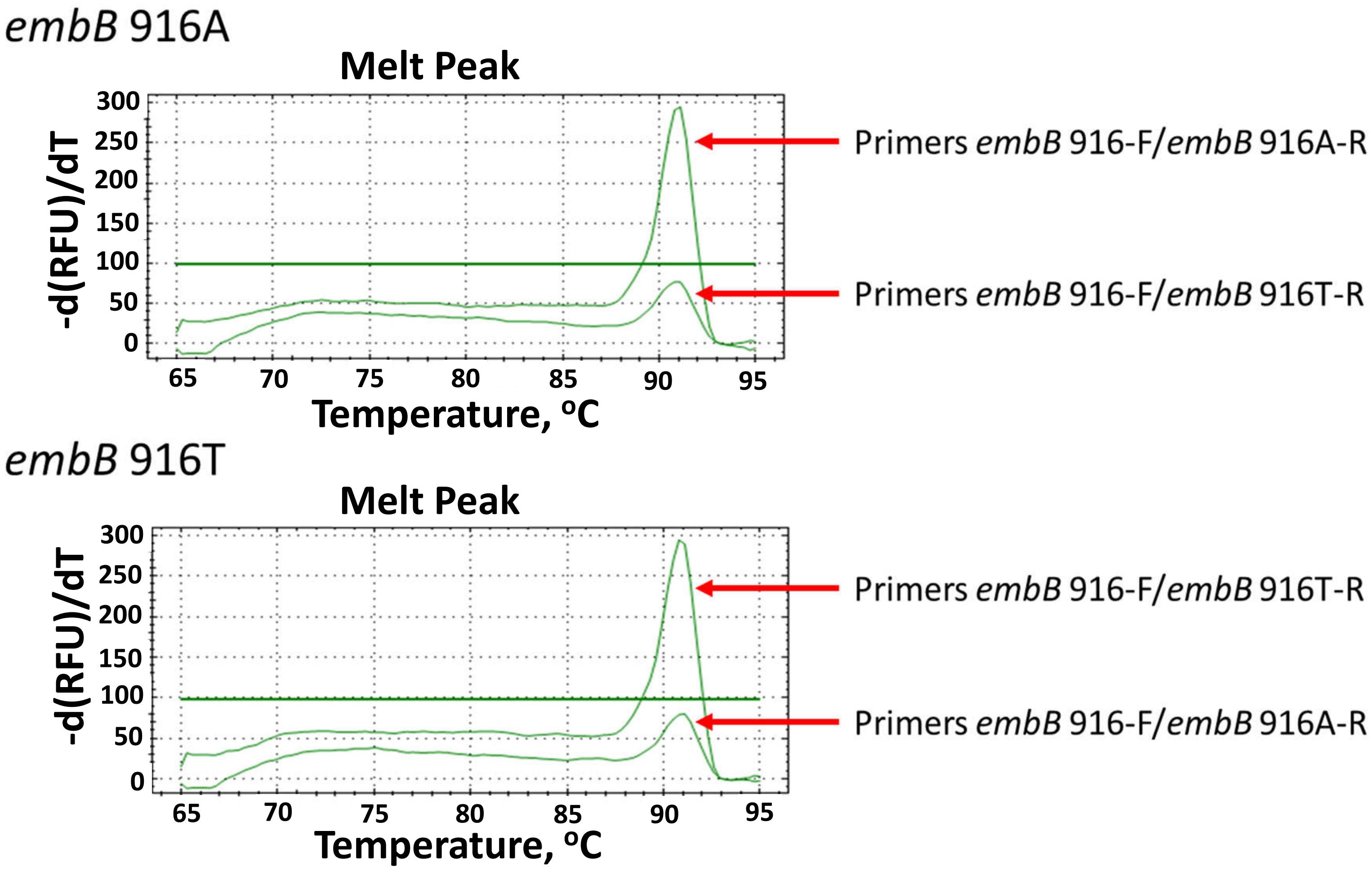
embB 916A
Melt Peak
-d(RFU)/dT
300
250
200
150
100
50
0
65
70
75
80
85
90
95
Temperature, °C
Primers embB 916-F/embB 916A-R
Primers embB 916-F/embB 916T-R
embB 916T
Melt Peak
-d(RFU)/dT
300
250
200
150
100
50
0
65
70
75
80
85
90
95
Temperature, °C
Primers embB 916-F/embB 916T-R
Primers embB 916-F/embB 916A-R
