# Supplementary material for: Rapid screening mutations of first-line-drug-resistant genes in Mycobacterium tuberculosis strains by allele-specific real-time quantitative PCR
Source: PeerJ. 2019 Apr 1;7:e6696. doi: 10.7717/peerj.6696 (PMC6448557; doi:10.7717/peerj.6696)
Supplement: Supplemental Information 4 [file peerj-07-6696-s004.docx]

Table S1. Information of AS-PCR primers.

| Primer name | Annealing temperature | Primer sequence (5′-3′) ^***^ | Product size | Detecting allele | Detection threshold |
| --- | --- | --- | --- | --- | --- |
| *KatG* 898G-F | 60 ^o^C ^**^ | AGCAGATGGGCTTGGACG | 156 bp | 898G  (condon 300) | 5 × 10^2^ |
| *KatG* 898-R |  | TCTTCGTCAGCTCCCACTC |  |  |  |
| *KatG* 898T-F |  | GAGCAGATGGGCTTGGACT | 157 bp | 898T  (condon 300) |  |
| *KatG* 898-R |  | TCTTCGTCAGCTCCCACTC |  |  |  |
| *KatG* 906A-F | 65 ^o^C | GGCTTGGGCTGGAAGTGA | 273 bp | 906A  (condon 302) | 10^4^ |
| *KatG* 906-R ^*^ |  | ATCCACCCGCAGCGAGAG |  |  |  |
| *KatG* 906C-F |  | GGCTTGGGCTGGAAGATC | 273 bp | 906C  (condon 302) |  |
| *KatG* 906-R ^*^ |  | ATCCACCCGCAGCGAGAG |  |  |  |
| *KatG* 944C-F | 65 ^o^C | TAAGGACGCGATCACGAC | 485 bp | 944C  (condon 315) | 5 × 10^2^ |
| *KatG* 944-R ^*^ |  | TGAGACAGTCAATCCCGATG |  |  |  |
| *KatG* 944G-F |  | GTAAGGACGCGATCACGAG | 486 bp | 944G  (condon 315) |  |
| *KatG* 944-R^*^ |  | TGAGACAGTCAATCCCGATG |  |  |  |
| *RpoB* 1576A-F | 55 ^o^C | CGCTGTCGGGGTTTACCA | 464 bp | 1576A  (condon 526) | 5 × 10^2^ |
| *RpoB* 1576-R |  | GTGCTCCAGGAAGGGAATCAT |  |  |  |
| *RpoB* 1576C-F |  | CGCTGTCGGGGTTGATCC | 464 bp | 1576C  (condon 526) |  |
| *RpoB* 1576-R |  | GTGCTCCAGGAAGGGAATCAT |  |  |  |
| *RpoB* 1576T-F | 60 ^o^C | CGCTGTCGGGGTTGTCCT | 464 bp | 1576T  (condon 526) | 5 × 10^2^ |
| *RpoB* 1576-R |  | GTGCTCCAGGAAGGGAATCAT |  |  |  |
| *RpoB* 1576C-F |  | CGCTGTCGGGGTTGATCC | 464 bp | 1576C  (condon 526) |  |
| *RpoB* 1576-R |  | GTGCTCCAGGAAGGGAATCAT |  |  |  |
| *RpoB* 1592T-F | 45 ^o^C | CCACAAGCGCCGACTCTT | 448 bp | 1592T  (condon 531) | 10^4^ |
| *RpoB* 1592-1R |  | GTGCTCCAGGAAGGGAATCAT |  |  |  |
| *RpoB* 1592C-F |  | CCACAAGCGCCGACAGTC | 446 bp | 1592C  (condon 531) |  |
| *RpoB* 1592-2R |  | GCTCCAGGAAGGGAATCAT |  |  |  |
| *RpoB* 1843A-F | 55 ^o^C | ACGAGGAGGACCGCCATA | 195 bp | 1843A  (condon 615) | 5 × 10^2^ |
| *RpoB* 1843-R |  | GCTCCAGGAAGGGAATCAT |  |  |  |
| *RpoB* 1843G-F |  | ACGAGGAGGACCGCTACG | 195 bp | 1843G  (condon 615) |  |
| *RpoB* 1843-R |  | GCTCCAGGAAGGGAATCAT |  |  |  |
| *RpsL* -6T-F | 60 ^o^C | CGCCCAAGATAGAACGCT | 400 bp | -6T | 10^3^ |
| *RpsL* -6-R |  | CATCAGCCCTTCTCCTTCTTAG |  |  |  |
| *RpsL* -6C-F |  | CGCCCAAGATAGAACGCC | 400 bp | -6C |  |
| *RpsL* -6-R |  | CATCAGCCCTTCTCCTTCTTAG |  |  |  |
| *RpsL* 128G-F | 60 ^o^C | TGTACACCACCACTGCGAG | 206 bp | 128G  (condon 43) | 5 × 10^2^ |
| *RpsL* 128-R |  | CTGCGTATCCAGCGAACC |  |  |  |
| *RpsL* 128A-F |  | TGTACACCACCACTGCGAA | 206 bp | 128A  (condon 43) |  |
| *RpsL* 128-R |  | CTGCGTATCCAGCGAACC |  |  |  |
| *RpsL* 263-F | 60 ^o^C | CACCACCACTCCGAAGAAGC | 167 bp | 263G  (condon 88) | 10^3^ |
| *RpsL* 263G-R |  | GCACACCAGGCAGGTCGC |  |  |  |
| *RpsL* 263-F |  | CTCCGAAGAAGCCGAACTC | 160 bp | 263A  (condon 88) |  |
| *RpsL* 263A-R |  | CGCACACCAGGCAGGTACT |  |  |  |
| *EmbB* 233-F | 55 ^o^C | GGGCTGATTGGCTTTGTG | 151 bp | 233G  (condon 78) | 10^3^ |
| *EmbB* 233G-R |  | GCACGGTGGCGGTAAAAC |  |  |  |
| *EmbB* 233-F |  | GGGCTGATTGGCTTTGTG | 151 bp | 233A  (condon 78) |  |
| *EmbB* 233A-R |  | GCACGGTGGCGGTAGAGT |  |  |  |
| *EmbB* 916-F | 60 ^o^C | ACCACGCTGAAACTGCTG | 291 bp | 916G  (condon 306) | 5 × 10^2^ |
| *EmbB* 916G-R |  | GTCGGCGACTCGGGCTAC |  |  |  |
| *EmbB* 916-F |  | ACCACGCTGAAACTGCTG | 291 bp | 916A  (condon 306) |  |
| *EmbB* 916A-R |  | GTCGGCGACTCGGGCTAT |  |  |  |
| *EmbB* 916-F | 65 ^o^C | ACCACGCTGAAACTGCTG | 291 bp | 916T  (condon 306) | 5 × 10^2^ |
| *EmbB* 916T-R |  | GTCGGCGACTCGGGCTAA |  |  |  |
| *EmbB* 916-F |  | ACCACGCTGAAACTGCTG | 291 bp | 916A  (condon 306) |  |
| *EmbB* 916A-R |  | GTCGGCGACTCGGGCTAT |  |  |  |
| *EmbB* 918C-F | 64 ^o^C | CGGCTACATCCTGGTCATC | 349 bp | 918C  (condon 306) | 10^3^ |
| *EmbB* 918-R ^*^ |  | ATAGGTGACCAGCGAGCC |  |  |  |
| *EmbB* 918G-F |  | GGCTACATCCTGGGCGTG | 196 bp | 918G  (condon 306) |  |
| *EmbB* 918-2R |  | ACAGCAGCAGCCAGCACA |  |  |  |
| *EmbB* 918A-F | 55 ^o^C | GGCTACATCCTGGGCACA | 196 bp | 918A  (condon 306) | 10^3^ |
| *EmbB* 918-2R |  | ACAGCAGCAGCCAGCACA |  |  |  |
| *EmbB* 918G-F |  | GGCTACATCCTGGGCGTG | 196 bp | 918G  (condon 306) |  |
| *EmbB* 918-2R |  | ACAGCAGCAGCCAGCACA |  |  |  |
| *EmbB* 1217-F | 60 ^o^C | GACGCCAGTCTGTGGATGC | 202 bp | 1217C  (condon 406) | 5 × 10^2^ |
| *EmbB* 1217C-R |  | AGCCGAGCGCGATGATAG |  |  |  |
| *EmbB* 1217-F |  | GACGCCAGTCTGTGGATGC | 202 bp | 1217G  (condon 406) |  |
| *EmbB* 1217G-R |  | AGCCGAGCGCGATGATAC |  |  |  |
| 16S 915-F | — | CGCACAAGCGGCGGAGCA | 104 bp | Inner control | — |
| 16S 1018-R |  | GCCACAAGGGAACGCCTATCT |  |  |  |

Note:

^*^ Primers *KatG* 906-R, *KatG* 944-R, and *EmbB* 918-R were replaced by *KatG* 898-R, *KatG* 898-R, and, *EmbB* 918-2R in real-time PCR, respectively.

^**^ Annealing temperature of real-time PCR is 67 ^o^C.

^***^ The factitious allele mutations in primers were marked in red. The primers with two factitious nucleotides were for mutant alleles, while the primers with one factitious nucleotide were for wild- type alleles.
