# Supplementary material for: Rapid screening mutations of first-line-drug-resistant genes in Mycobacterium tuberculosis strains by allele-specific real-time quantitative PCR
Source: PeerJ. 2019 Apr 1;7:e6696. doi: 10.7717/peerj.6696 (PMC6448557; doi:10.7717/peerj.6696)
Supplement: Supplemental Information 5 [file peerj-07-6696-s005.docx]

Table S2. Information for primers used to construct plasmids.

| Primer | Annealing temperature | Primer sequence (5′-3′) | Product size |
| --- | --- | --- | --- |
| *KatG* 549-F | 55 ^o^C | CCGCCTTTGCTGCTTTCTC | 983 bp |
| *KatG* 1531-R |  | GGGGCTGATCTACGTGAAC |  |
| *RpoB* 925-F | 55 ^o^C | GTCACCGTGCTGCTCAAG | 1295 bp |
| *RpoB* 2219-R |  | CCGTTGTCGTGCATCACAG |  |
| *RpsL* 44-F2 | 55 ^o^C | ATGAGACGAATCGAGTTTGAGG | 640 bp |
| *RpsL* 683-R2 |  | GATCGGTGCCGGTCTTGTCG |  |
| *EmbB* 318-F | 55 ^o^C | CTGCCAGCGACCGTTTTC | 645 bp |
| *EmbB* 327-R |  | GGCGTCCTTGCCTTGCTT |  |
| *EmbB* 333-F2 | 55 ^o^C | GCAGGCGTTGTTCGTCG | 1442 bp |
| *EmbB* 1774-R2 |  | ACTTGGTGGGCGTGAACAT |  |
